# Familial Hyperaldosteronism Type 3 with a Rapidly Growing Adrenal Tumor: An In Situ Aldosterone Imaging Study

**DOI:** 10.3390/cimb44010010

**Published:** 2021-12-28

**Authors:** Nae Takizawa, Susumu Tanaka, Koshiro Nishimoto, Yuki Sugiura, Makoto Suematsu, Chisato Ohe, Haruyuki Ohsugi, Yosuke Mizuno, Kuniaki Mukai, Tsugio Seki, Kenji Oki, Celso E. Gomez-Sanchez, Tadashi Matsuda

**Affiliations:** 1 Department of Urology and Andrology, Kansai Medical University, Osaka 573-1191, Japan; yamamotn@hirakata.kmu.ac.jp (N.T.); haosugi@gmail.com (H.O.); matsudat@takii.kmu.ac.jp (T.M.); 2 Department of Anatomy, Kansai Medical University, Osaka 573-1191, Japan; tanakass@hirakata.kmu.ac.jp; 3 Department of Uro-Oncology, Saitama Medical University, International Medical Center 1397-1 Yamane, Hidaka 350-1298, Japan; 4 Departments of Biochemistry, School of Medicine, Keio University, Tokyo 160-8582, Japan; yuki.sgi@gmail.com (Y.S.); gasbiology@keio.jp (M.S.); k-mukai@keio.jp (K.M.); 5 Department of Pathology, Kansai Medical University, Osaka 573-1191, Japan; ohec@hirakata.kmu.ac.jp; 6 Biomedical Research Center, Division of Morphological Science, Saitama Medical University, Saitama 350-0495, Japan; mizuno@saitama-med.ac.jp; 7 Department of Medical Education, California University of Science and Medicine, Colton, CA 92324, USA; SekiT@cusm.org; 8 Department of Molecular and Internal Medicine, Graduate School of Biomedical and Health Sciences, Hiroshima University, Hiroshima 734-8551, Japan; kenjioki@hiroshima-u.ac.jp; 9 Medical Service, G.V. (Sonny) Montgomery VA Medical Center, Department of Pharmacology and Toxicology, Medicine, University of Mississippi Medical Center, Jackson, MS 39216, USA; cgomez-sanchez@umc.edu

**Keywords:** familial hyperaldosteronism type 3, *KCNJ5*, adrenal tumor, β-catenin, MALDI-IMS, CYP11B2

## Abstract

Primary aldosteronism is most often caused by aldosterone-producing adenoma (APA) and bi-lateral adrenal hyperplasia. Most APAs are caused by somatic mutations of various ion channels and pumps, the most common being the inward-rectifying potassium channel *KCNJ5*. Germ line mutations of *KCNJ5* cause familial hyperaldosteronism type 3 (FH3), which is associated with severe hyperaldosteronism and hypertension. We present an unusual case of FH3 in a young woman, first diagnosed with primary aldosteronism at the age of 6 years, with bilateral adrenal hyperplasia, who underwent unilateral adrenalectomy (left adrenal) to alleviate hyperaldosteronism. However, her hyperaldosteronism persisted. At the age of 26 years, tomography of the remaining adrenal revealed two different adrenal tumors, one of which grew substantially in 4 months; therefore, the adrenal gland was removed. A comprehensive histological, immunohistochemical, and molecular evaluation of various sections of the adrenal gland and in situ visualization of aldosterone, using matrix-assisted laser desorption/ionization imaging mass spectrometry, was performed. Aldosterone synthase (CYP11B2) immunoreactivity was observed in the tumors and adrenal gland. The larger tumor also harbored a somatic β-catenin activating mutation. Aldosterone visualized in situ was only found in the subcapsular regions of the adrenal and not in the tumors. Collectively, this case of FH3 presented unusual tumor development and histological/molecular findings.

## 1. Introduction

Primary aldosteronism (PA) is caused by excessive and autonomous secretion of aldosterone and is classified with aldosterone-producing adenoma (APA), bilateral idiopathic hyperaldosteronism (IHA), unilateral hyperplasia, or aldosterone-producing carcinoma. Somatic mutations in ion channel/pump genes, including the inwardly rectifying subfamily J, member 5 potassium channel (*KCNJ5*), have been identified in a significant percentage of APAs (APA-associated mutations) [1]. *KCNJ5* mutations cause a loss in specificity of the channel’s selectivity filter for potassium. This leads to sodium leakage into the cells, causing depolarization of the membrane potential; this results in increased calcium influx into adrenocortical cells, causing autonomous aldosterone production [1]. There are four types of familial hyperaldosteronism (FH1–FH4) [2]. FH3 is caused by a germline mutation of *KCNJ5* that leads to adrenal hyperplasia with a marked increase in the secretion of aldosterone [1]. We and others have recently reported cases of non-familial juvenile PA due to mosaicism of somatic *KCNJ5*-mutated and non-mutated cells [3,4], in which the mutated cells/tissues were hyperplastic.

We previously described an immunohistochemistry protocol for aldosterone synthase (CYP11B2) that distinguishes CYP11B2 from the cortisol-synthesizing enzyme steroid 11β-hydroxylase (CYP11B1) [5]. Using CYP11B2 staining, putative aldosterone-producing cells were visualized in the zona glomerulosa of normal adrenals from infants and adults [5,6,7], as well as several PA lesions [4,5,8]. However, aldosterone biosynthesis requires a cascade of steroidogenic enzymes, and the presence of CYP11B2 alone is not sufficient for the synthesis of aldosterone. To visualize the aldosterone localization in adrenal sections, we recently developed a protocol for the in situ detection of aldosterone using state-of-the-art matrix-assisted laser desorption/ionization imaging mass spectrometry (MALDI-IMS) [9]. Since the steroid hormones, including aldosterone, are released into the blood stream immediately after production, i.e., there is no intracellular storage of steroid hormones, the detection of aldosterone in cells using MALDI-IMS indicates that those cells are actively producing aldosterone. In the present study, we describe an FH3 case with results of comprehensive molecular and CYP11B2 immunohistochemical analyses and correlated them with aldosterone localization in adrenal tissue.

## 2. Case

**Figure 1:**
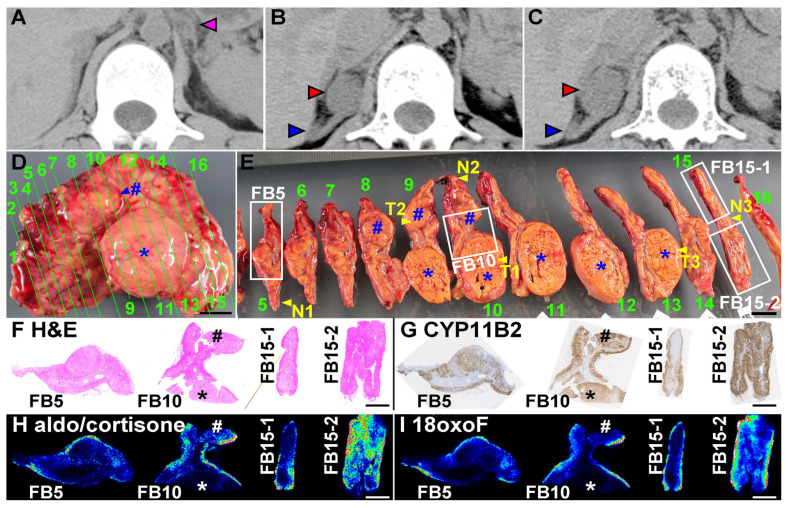
CT and histological findings of the case. (**A**) CT findings at 15 years of age. The left adrenal gland was removed after CT examination. (**B**) CT findings at 26 years of age. Red and blue arrowheads indicate the larger and smaller adrenal tumors in the right adrenal gland, respectively. (**C**) CT findings 4 months after the CT shown in panel B. The larger tumor significantly enlarged in 4 months. (**D**) Macroscopic findings of the extracted right adrenal. The larger (*) and the smaller (#) tumors presumably corresponded to the large (red arrowhead) and small (blue arrowhead) tumors in panels (B) and (**C**), respectively. The adrenal was cut into 16 pieces at the green lines. (**E**) Cut surfaces of the extracted adrenal. The green numbers in panel (**D**) correspond to the numbers in panel (**E**). The cut surface numbers in panels (**D**,**E**) correspond to those in parentheses in Supplementary Figure S1, which shows the sections after formaldehyde fixation. Frozen tissue blocks, in an optimal cutting temperature compound, were prepared from 4 portions, indicated by white frames (FB5, FB10, FB15-1, and FB15-2). Flash frozen tissues were also taken from 3 non-tumor portions (N1–N3) and 3 tumor portions (T1–T3). (**F**–**I**) Hematoxylin and eosin staining, immunohistochemistry for CYP11B2, MALDI-imaging of aldosterone and cortisone (aldo/cortisone), and that of 18-oxocortisol (18oxoF), respectively, of frozen tissues.

We present the case of a 27-year-old Japanese female with a history of severe juvenile PA. She was diagnosed with PA due to bilateral adrenal hyperplasia following adrenal vein sampling at the age of six years and treated with spironolactone and potassium supplementation with moderate control of her blood pressure [10]. However, when she was 15 years old, her serum creatinine level increased to 2.04 mg/dL (normal range: 0.4–1 mg/dL) due to severe hypertension and persistent high plasma aldosterone concentration (PAC: 2511 pg/mL (normal range: 35.7–240 pg/mL)). Since multiple adrenal vein catheterization attempts failed, and computed tomography (CT) indicated that her left adrenal gland (pink arrowhead in Figure 1A) was more hyperplastic than the right, she underwent left adrenalectomy, expecting to alleviate hyperaldosteronism. However, although lower than before, PAC remained elevated (1280 pg/mL) after surgery. At the age of 21 years, she developed end-stage chronic renal failure, thereby requiring intermittent hemodialysis. When she was 26 years old, a CT detected two adrenal tumors (22 × 17 mm and 10 × 6 mm) in her right adrenal gland (red and blue arrowheads in Figure 1B, respectively). Four months later, the larger tumor grew further (28 × 25 mm), and the smaller tumor remained unchanged (Figure 1C), suggesting that the larger tumor might be an adrenocortical carcinoma. She underwent a laparoscopic right adrenalectomy with removal of the intact gland in toto. Her PAC fell into the normal range (83 pg/mL) while on replacement with prednisolone for bilateral adrenalectomy.

## 3. Materials and Methods

### 3.1. DNA and RNA Isolation from Flash Frozen Tissues, Blood, and Hair Root

Using the AllPrep DNA/RNA Mini Kit (catalog#: 80204, Qiagen, Valencia, CA, USA) and ISOHAIR (catalog#: 315-3403, NIPPON GENE CO., LTD., Tokyo, Japan), genomic DNA and RNA (DNA/RNA) #86, 87, 88, 89, 90, and 91 were prepared from N1, N2, N3, T1, T2, and T3, respectively, as previously reported [11]. DNA #92, 93, 155, and 156 were isolated from the patient’s blood, mother’s blood, patient’s hair root, and father’s blood, respectively, according to the manufacturer’s instruction.

### 3.2. Whole Exome Sequencing

We performed whole exome sequencing of genomic DNA samples from T1, N1, and blood (Bl), which was carried out at RIKEN GENESIS CO., LTD. (Tokyo, Japan), as follows. DNA was sheared into approximately 200 bp fragments and used to construct a library for multiplexed paired-end sequencing with the SureSelectXT Reagent Kit (catalog#: G9641B, Agilent Technologies, Santa Clara, CA, USA). The constructed library was hybridized to biotinylated cRNA baits from the SureSelectXT Human All Exon V6 Kit (catalog#: 5190–8865, Agilent Technologies, Santa Clara, CA, USA) for target enrichment. Targeted sequences were purified with magnetic beads, amplified, and sequenced on an Illumina HiSeq 2500 platform in paired-end 101 bp configuration.

The raw sequence read data of the three samples passed the quality checks in FASTQC (https://www.bioinformatics.babraham.ac.uk/projects/fastqc/ (accessed on 1 February 2017)). Read trimming via base quality was performed using Trimmomatic [12]. Read alignment was performed with the Burrows–Wheeler Aligner [13] (version 0.7.15-r1140). hs37d5 was used as the reference human genome. PCR duplicate reads were removed using Picard (version 2.9.0-1-gf5b9f50-SNAPSHOT, https://broadinstitute.github.io/picard/ (accessed on 1 February 2017)). Non-mappable reads were removed using SAMtools (version 1.3.1) [14]. After filtering out those reads, we applied the Genome Analysis Toolkit [15] (GATK version 3.5-0-g36282e4) base quality score recalibration and performed SNP and INDEL discovery (HaplotypeCaller). Finally, we identified 350, 346, and 343 variants in samples T1, N1, and Bl, respectively, and the variants were annotated using ANNOVAR (version 2016Feb1) [16]. As expected, the *KCNJ5* (p.G151R) mutation was identified in these three samples.

Variants that passed quality control were prioritized according to the following strategies. We only retained variants predicted to modify protein function; these included the nonsense, splice site, coding indel, and missense variants. We removed variants with minor allele frequencies >0.4% for the ESP6500 (ESP6500siv2_all provided by ANNOVAR) database, >0.4% for each population of the Exome Aggregation Consortium (exac03 provided by ANNOVAR), >0.4% in HGVD (containing genetic variations determined by exome sequencing of 1208 individuals in Japan) [17], and >0.4% in 2KJPN (whole-genome sequences of 2049 Japanese healthy individuals and construction of a highly accurate Japanese population reference panel). After removing these variants, we focused on variants identified only in the tumor sample. Variants that appeared to be mapping artifacts, and were too common in in-house controls, were also excluded from further analyses. Consequently, several somatic mutations were found in sample T1. Sanger sequencing of these genes confirmed mutations in catenin β 1 (*CTNNB1*), centromere protein E (*CENPE*), leucine zipper- and EF-hand-containing transmembrane protein 2 (*LETM2*), and ALG10 Alpha-1,2-Glucosyltransferase B (*ALG10B*) in T1 and T3, but not in T2, N1–N3, and Bl, suggesting that these genes might be associated with the rapid growth of the larger tumor. Among these genes, it is well known that mutation in *CTNNB1* is associated with tumor growth in adrenocortical carcinoma via the constitutively activated nuclear β catenin protein [18].

### 3.3. Microarray Analyses

Microarray analyses of T1–T3 (RNA#89–91, respectively) and N1–N3 (RNA#86 – 88) were performed using the Human Clariom™ S Array and GeneChip WT PLUS Reagent Kit (Thermo Fisher Scientific, #902916 and 902280) [7]. N1 was presumably contaminated with cells from the adrenal medulla, because a few genes known to be expressed in the adrenal medulla were highly expressed in N1 samples (e.g., tyrosine hydroxylase). Genes that exhibited a fold change of 1.3 or more in T1 and T3, as compared to N2 and N3, were used for pathway analysis using the Kyoto Encyclopedia of Genes and Genomes Database. Six pathways were significantly identified as upregulated, as follows: “Protein digestion and absorption” (*p* = 0.0016), “Renin-angiotensin system” (*p* = 0.0023), “Adipocytokine signaling pathway” (*p* = 0.0058), “Cell cycle” (*p* = 0.0091), “Pancreatic secretion” (*p* = 0.0171), and “p53 signaling pathway” (*p* = 0.0339). A similar analysis, using the WikiPathway Database, revealed three up-regulated pathways, as follows: “Retinoblastoma Gene in Cancer” (*p* = 0.0003), “Splicing factor NOVA regulated synaptic proteins” (*p* = 0.0091), and “Deregulation of Rab and Rab Effector Genes in Bladder Cancer” (*p* = 0.0114). Upstream steroidogenic enzymes for aldosterone synthesis (i.e., cytochrome p450 family 11 subfamily A member 1 (CYP11A1), 3-β-hydroxysteroid dehydrogenase (HSD3B2), and 21-hydroxylase (CYP21A1)) did not exhibit variation in expression between samples, suggesting that the localization of aldosterone shown by MALDI-IMS (mainly in the subcapsular area but not in the tumors) was not due to a lack of the upstream steroidogenic enzyme expression, but other unidentified reasons. Overall, the status of gene variants and gene expression was consistent with the clinical course of the case.

### 3.4. Confirmation of CYP11B2 Expression

We compared the expression levels of *CYP11B2* mRNA between this case and archived APA cases, previously adrenalectomized in the Kansai Medical University (APA#7–APA#26), using qRT-PCR for *CYP11B2* (Supplementary Table S1). *CYP11B2*-expression levels in the tumors of cases APA#7, 18, 19, and 26 were lower than those in paired adjacent adrenal tissues, suggesting incorrect sampling or sampling from non-APA tumors; therefore, these samples were removed from the following analyses. Two tumors (tumors 1 and 2) were sampled from APA#9, but the *CYP11B2* expression of tumor 2 was lower than that of adjacent normal; therefore, tumor 2, but not tumor 1, was also removed from the following analyses. Normal adrenal APA#23 (APA#23N) showed the lowest *CYP11B2* expression level, and a fold difference of each sample (16 pairs) over APA#23N was calculated. Sanger sequencing of these cases for *KCNJ5* revealed that 10 cases harbored *KCNJ5* mutations (62.5%, p.G151R [*n* = 6], and p.L168R [*n* = 3], p.L168Hfs*93 [*n* = 1]) in samples from tumors, but not in their paired adjacent adrenals. An average fold change of APA samples with *KCNJ5* mutation (513,214.9 ± 290,452.9 [mean ± S.D.]) was similar to that without *KCNJ5* mutation (708,321.1 ± 297,156.8, *p* = 0.319, unpaired Student’s *t*-test using ΔΔCt values). As expected from the results of CYP11B2 immunohistochemistry, *CYP11B2* expression levels were not different between non-tumor (816,286.5 ± 428,233.7-fold) and tumor portions (539,737.3 ± 336,381.8-fold) of the case (*p* = 0.393, unpaired Student’s *t*-test using ΔΔCt values). *CYP11B2* expression levels in the case (T1–T3 and N1–N3, 582,237 [interquartile range: 448,734–977,189]-fold) and APA (574,401 (328,933–817,145)-fold) were significantly higher than that of the paired adjacent normal adrenals (966 (62–11,986)-fold), and those in the case and APA were similar (Supplementary Figure S6). Consequently, whole enlarged adrenal in the case expressed high levels of CYP11B2 in mRNA and protein as APAs did.

## 4. Result

### 4.1. Analyses of the Surgically Removed Adrenal Gland

Comprehensive pathological and molecular analyses were approved by the institutional review boards. Immediately after surgery, the adrenal gland was cut into 16 pieces, as shown in Figure 1D,E. The adrenal gland had two apparent tumors (# and * in Figure 1D,E) and many smaller nodules. Flash frozen tissues were also taken from three non-tumor portions (N1–N3 in Figure 1E), two portions from the larger tumor (T1 and T3), and one portion from the smaller tumor (T2). Frozen blocks, embedded in optimal cutting temperature compound, were prepared from four portions (FB5, FB10, FB15-1, and FB15-2 in Figure 1E), as previously reported [7,9]. The remaining adrenal tissues were fixed with 10% formalin (Supplementary Figure S1) and used for formalin-fixed paraffin-embedded (FFPE) blocks for regular pathological diagnosis. Sanger sequencing of *KCNJ5* was performed, as previously reported [11], and a de novo *KCNJ5* mutation (p.G151R) was detected in genomic DNA from N1–N3 (DNA #86–88, respectively), T1–T3 (#89–92, respectively), as well as her blood (Blood, #92) and hair root (#155), but not in blood samples from her mother (#93) and father (#156) (see “DNA and RNA isolation from flash frozen tissues, blood, and hair root” in the Materials and Methods, Supplementary Figure S2, and details of DNA samples shown in Supplementary Table S1). Histological analyses, using the frozen blocks (Figure 1F and Figure S3) and FFPE tissues (Supplementary Figure S4), were performed. Microscopically, most of the tumor cells in the larger and smaller tumors (* and # in Figure 1D–E, respectively) were composed of compact cells and lipid-rich cells but did not fulfill the criteria for adrenocortical carcinoma (Weiss score: 2; Ki-67 proliferation index: 4.0%), leading to the diagnosis of an adrenal adenoma. The adjacent non-tumor portion lost typical adrenocortical zonation in hematoxylin and eosin staining and revealed many cells that contained lipid vacuoles (Figure 1F, Supplementary Figures S3 and S4). CYP11B2 immunohistochemistry confirmed that the tumors were APAs, as CYP11B2 was expressed throughout the tumors (* and # in Figure 1G) [5]. The cortex of the adjacent non-tumor portion had many CYP11B2-positive cells with irregular arrangement, similar to the adrenal cortices of the previously removed left adrenal gland (Supplementary Figure S5) and as in a previously reported FH3 case [8].

### 4.2. Production of Aldosterone in FH3 Adrenal

We performed MALDI-IMS, using SolariX attached with Fourier transform ion cyclotron resonance mass spectrometry (Bruker Daltonics, Billerica, MA, USA), to demonstrate in situ aldosterone production throughout the adrenal, as we previously reported [9]. Aldosterone and cortisone, which share identical mass-to-charge ratio values (m/z), were identified mainly in the subcapsular areas of non-tumor adrenal gland, but not in tumors (Figure 1H), irrespective of strong CYP11B2 expression throughout the non-tumor adrenal gland and adrenal tumors (Figure 1G). The hybrid steroid 18-oxo-cortisol, a steroid marker of aldosterone-producing cells [9], was similarly detected in the subcapsular area only (Figure 1I). To determine the CYP11B2 mRNA levels in various areas of the adrenal, including the APA and adjacent adrenal cortices to the APA, we performed quantitative real-time polymerase chain reaction (qRT-PCR), as previously reported [4,7,11,19]. We confirmed that the CYP11B2 expression levels were not significantly different between the tumor and non-tumor portions (T1 – T3 and N1 – N3, respectively) (ΔCT in Supplementary Table S1, *p* = 0.303, Student’s *t*-test). In addition, there was no significant difference in the expression level of CYP1B2 between the case, i.e., the average of T1–T3 and N1–N3, and unrelated archived cases of sporadic APA (*n* = 16, Supplementary Figure S6, data of the archived APA cases are shown in Supplementary Table S1). Immunohistochemistry of KCNJ5 was performed in frozen sections obtained from a normal adrenal gland of a renal cell carcinoma patient (left in Supplementary Figure S7A) and FB10 (right), as previously reported [19]. KCNJ5 was detected only in the subcapsular area of the normal adrenal tissue, as previously reported (Supplementary Figure S7B) [19]; whereas, in this case, it was found throughout the adrenal cortex and tumors (Supplementary Figure S7C,D), suggesting that the *KCNJ5* mutation induced KCNJ5 and CYP11B2 co-expression throughout the adrenal cortex and tumors in the patient. Irrespective of high levels of CYP11B2 and KCNJ5, the tumors produced much lower levels of aldosterone and 18oxoF than the non-tumor portion, as shown in Figure 1H,I.

Whole exome sequencing confirmed a germline mutation of *KCNJ5* in T1, N1, and the patient’s blood (Materials and Methods). Several somatic mutations were identified in the larger tumor, T1, but not in N1 and blood, which included β-catenin (*CTNNB1*, c.134C > A, p.S45Y), *ADAM17*, *CENPE*, *COL12A1*, *LETM2*, *ALG10B*, and *SRCAP*. Among these mutations, the *CTNNB1* mutation presumably caused rapid tumor growth. Immunohistochemistry confirmed nuclear CTNNB1 expression in the tumor but not in the non-tumor portions, suggesting activation of CTNNB1 in the tumor (Supplementary Figure S8). Microarray analyses of T1–T3 (RNA#89 – 91, respectively) and N1–N3 (RNA#86 – 88, respectively) were performed, as previously reported [7], and confirmed that genes of the cell proliferation pathway were upregulated in T1 and T3 (* in Supplementary Table S2). Except for hydroxysteroid 17-β-dehydrogenase 14 (HSD17B14), which is not associated with aldosterone synthesis, expression of steroidogenic enzymes did not differ between the aldosterone-negative tumors (T1–T3) and non-tumor portions (N1–N3), suggesting that aldosterone production in the non-tumor subcapsular area was controlled by other factor(s) than the steroidogenic enzymes, including CYP11B2.

### 4.3. Cellular Progression in Non-Tumor and Tumor Portions of the Case

**Figure 2:**
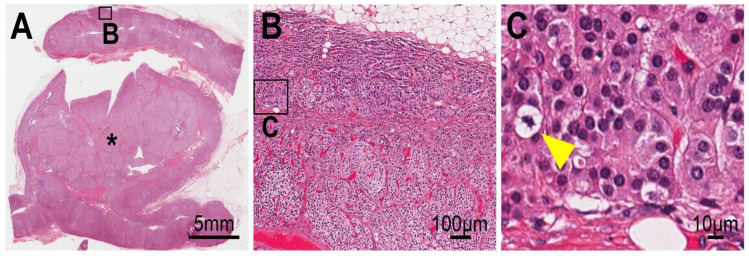
Adrenal histology of the case. The non-tumor adrenal portions were hyperplastic (panels (**A**) and (**B**)) and harbored mitotic cells (yellow arrowhead in panel (**C**)). *: the larger tumor (* in Figure 1).

**Figure 3:**
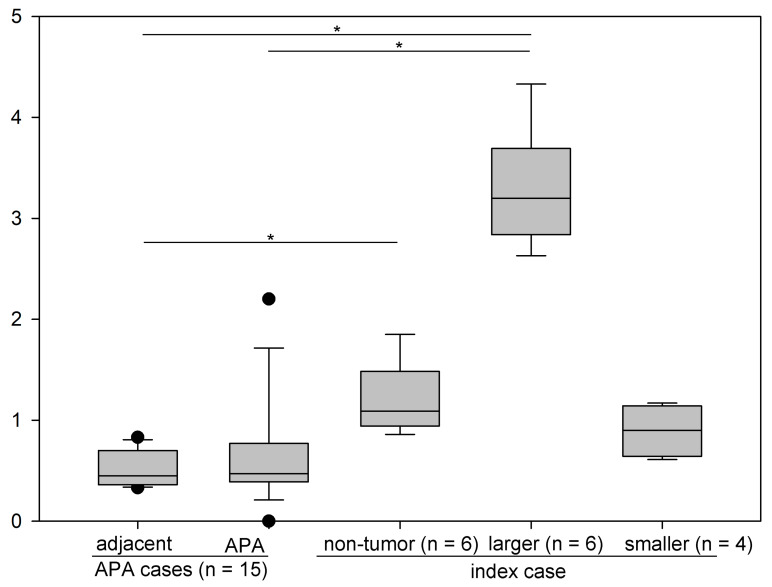
Comparison of Ki-67 index among APA cases (*n* = 15 each) and index case. * *p* < 0.05.

The non-tumor adrenal gland was hyperplastic (Figure 2A,B) and harbored mitotic cells (yellow arrowhead in Figure 2C). To assess the cell cycle progression status of the adrenal cells of the patient, we compared the Ki-67 index [20] between the non-tumor portions, the larger tumor (* in Figure 1 and Figure 2), smaller tumor (# in Figure 1), and archived sporadic APAs and their adjacent adrenal sections (“adjacent”) in cases APA #8, 10–17, and 20–25 (*n* = 15) in Supplementary Table S1. It is noteworthy that APA #9 was removed from the analysis because the case harbored a non-APA tumor (sample name: KS-APA_9_T2, Supplementary Table S1). Non-tumor portions were analyzed using two parts each from FFPE blocks #4, 10, and 14 (*n* = 6, Supplementary Figure S4). The larger tumor was analyzed using two parts each from FFPE block #9, 10, and 14 (*n* = 6). The smaller tumor was analyzed using four parts from FFPE block #8 (*n* = 4). Upon comparing these five groups (Kruskal–Wallis one-way analysis of variance on ranks, followed by post hoc comparison with Dunn’s methods), we found that the larger tumor (3.20 [interquartile range: 2.84–3.70] unit) had a higher index than the APAs (0.47 [0.39–0.77] unit, *p* = 0.001) and their adjacent adrenal tissue (0.45 [0.36–0.70] unit, *p* < 0.001) (Figure 3). Interestingly, the non-tumor portion of the patient showed a higher Ki-67 index (1.09 [0.94–1.48] unit) than the adjacent adrenal tissue in sporadic APAs (*p* = 0.047). These results suggest that chronic stimulation from the mutated *KCNJ5* channel and/or high aldosterone concentration around the cells might be associated with increased cell cycle progression and/or second hit mutations in genes, including *CTNNB1*.

## 5. Discussion

This is a case of FH3 with unusual tumor development and histological/molecular findings. The patient was initially diagnosed with PA at the age of 6 years, and her adrenals were removed at the age of 15 years (left adrenal, to alleviate hyperaldosteronism) and at the age of 27 years (right adrenal, due to an enlarging tumor on CT). Clinical data of this patient from birth to 14 years old, including those of kidney biopsy at 10 years old, were described in a preceding article [10], and those from the age of 14 years are provided in Supplementary Table S3. Comprehensive pathological and molecular analyses of the removed right adrenal and blood resulted revealed FH3. The findings can be summarized as follows: (i) an abnormal cellular arrangement of CYP11B2-positive cells throughout the adrenal gland, similar to observed in a previously reported FH3 case [8]; (ii) limited localization of aldosterone production, primarily in the non-tumorous sections, with widespread and very strong CYP11B2 expression in the tumor areas; (iii) rapid tumor growth, which may represent an early stage of adrenocortical carcinoma, caused presumably by second hit mutation of *CTNNB1* (p.S45Y) in *KCNJ5* mutated cells of the larger tumor. The presence of a β-catenin mutation causing constitutive activation of adrenal cell growth has been shown to induce adrenal hyperplasia and adrenal cancer development in mice [21].

There were several peculiar aspects in this case. The pathological findings were remarkably similar to those of previously reported cases [22,23], but with the development of adrenal tumors. The *KCNJ5* mutation, absent in the parents of the patient, represents a de novo mutation. In this case, the larger adrenal tumor also had a second mutation of *CTNNB1,* which led to accelerated cellular growth. Furthermore, even though the patient underwent a bilateral adrenalectomy, serum aldosterone was still detectable and within the normal range, suggesting the presence of extra-adrenal adrenocortical cells harboring the *KCNJ5* mutation, causing aldosterone production [24].

While CYP11B2 staining has been used as a marker for aldosterone production, in the current case, in situ imaging of aldosterone revealed that aldosterone was only found in the non-tumorous areas of the distorted adrenal, which is an additional peculiar finding. Microarray and qRT-PCR studies revealed that all steroidogenic enzymes responsible for aldosterone synthesis were present in sufficient quantities to lead to the increased production of aldosterone in both the tumorous and non-tumorous areas. However, aldosterone production did not occur in the tumorous areas. It has been reported that *KCNJ5* mutations result in depolarization of the adrenal cells with stimulation of calcium mobilization and calmodulin phosphorylation, resulting in transcriptional induction of steroidogenic enzymes [1]. The fact that the tumors exhibited increased levels of steroidogenic enzymes suggests that there is an additional deficiency (or deficiencies) in the steps required for aldosterone biosynthesis in this case. For the synthesis of aldosterone, in addition to transcription of steroidogenic enzymes, cholesterol transporters to mitochondria, such as steroidogenic acute regulatory protein (StAR), are also needed. In addition, StAR must be phosphorylated to exert its action. We speculate that phosphorylation of StAR was repressed, as microarray analysis revealed that StAR expression was not downregulated (Supplementary Table S2).

Although some authors have reported that a treatment with mineralocorticoid receptor antagonist controls hyperaldosteronism well in FH3, most cases in FH3 needed bilateral adrenalectomy for management of severe hyperaldosteronism [1,23]. We had held off a decision of bilateral adrenalectomy because we preferred to avoid lifelong glucocorticoid replacement therapy and the risk of adrenal crisis from bilateral adrenalectomy, if possible. However, that led to the chronic kidney disease and, ultimately, maintenance dialysis at an early age. Notably, this case suggested that chronic stimulation from *KCNJ5* mutation and/or severe hyperaldosteronism contribute to tumorigenic transformation. Therefore, we believe that FH3 patients should undergo bilateral adrenalectomy as soon as possible before irreversible organ damages occur, if mineralocorticoid receptor antagonists cannot control patients’ blood pressure.

## 6. Conclusions

This is an interesting case of a de novo germline mutation of the *KCNJ5* gene that resulted in severe hyperaldosteronism with serious target organ damage, resulting in end-stage renal disease. Steroidogenic and molecular studies of the adrenal gland demonstrated discrepancies that need further investigation.

## Data Availability

The data presented in this study are available on request from the corresponding author. The data are not publicly available due to privacy and ethical concerns.

## Supplementary Materials

The following are available online at https://www.mdpi.com/article/10.3390/cimb44010010/s1, Figure S1: The sections after formaldehyde fixation, Figure S2: Pedigree of the index patient and the results of Sanger sequencing, Figure S3: The original microscopic imaging data, Figure S4: HE and IHC of FFPE tissue sections, Figure S5: HE and IHC of FFPE tissue sections of previously resected adrenal gland, Figure S6: CYP11B2 expression, Figure S7: *KCNJ5* immunostaining, Figure S8: CTNNB1 immunostaining, Table S1: Clinical data, qPCR data, and *KCNJ5* mutation analysis of the index case, the parents of the index case, and other APA cases, Table S2: Microarray data, Supplementary Table S3: clinical course from 15 years old.
