# Supplementary material for: Familial Hyperaldosteronism Type 3 with a Rapidly Growing Adrenal Tumor: An In Situ Aldosterone Imaging Study
Source: Curr Issues Mol Biol. 2021 Dec 28;44(1):128–38. doi: 10.3390/cimb44010010 (PMC8929039; doi:10.3390/cimb44010010)
Supplement: Supplementary file 1 [file cimb-44-00010-s001.zip › Figure_S2.pdf]

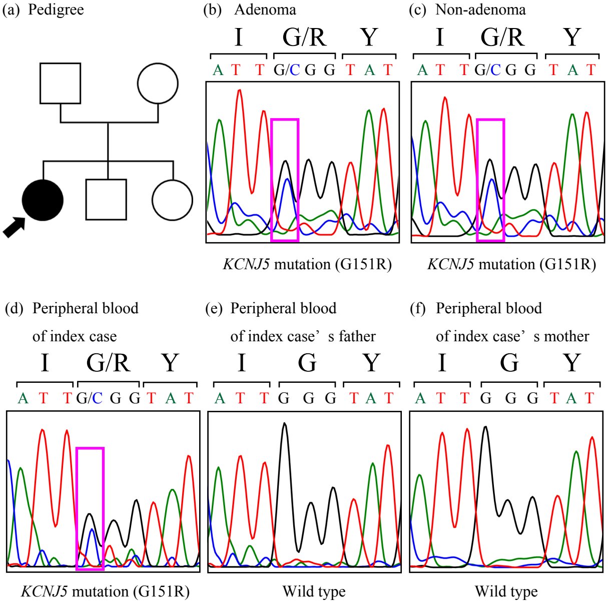
(a) Pedigree
(b) Adenoma
I G/R Y
A T T G/C G G T A T
KCNJ5 mutation (G151R)
(c) Non-adenoma
I G/R Y
A T T G/C G G T A T
KCNJ5 mutation (G151R)
(d) Peripheral blood of index case
I G/R Y
A T T G/C G G T A T
KCNJ5 mutation (G151R)
(e) Peripheral blood of index case’s father
I G Y
A T T G G G T A T
Wild type
(f) Peripheral blood of index case’s mother
I G Y
A T T G G G T A T
Wild type
