# Supplementary material for: Familial Hyperaldosteronism Type 3 with a Rapidly Growing Adrenal Tumor: An In Situ Aldosterone Imaging Study
Source: Curr Issues Mol Biol. 2021 Dec 28;44(1):128–38. doi: 10.3390/cimb44010010 (PMC8929039; doi:10.3390/cimb44010010)
Supplement: Supplementary file 1 [file cimb-44-00010-s001.zip › Figure_S4. FFPE section staining.pdf]

Figure S4

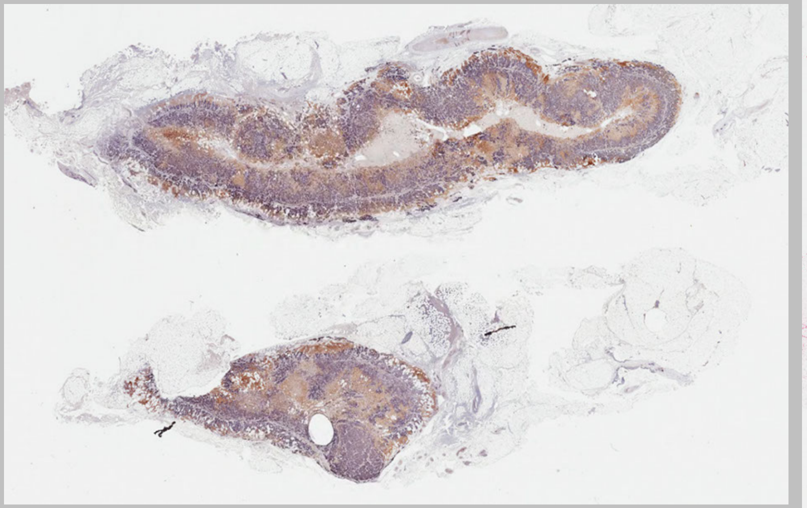

block 1-2, B2B1, 42713

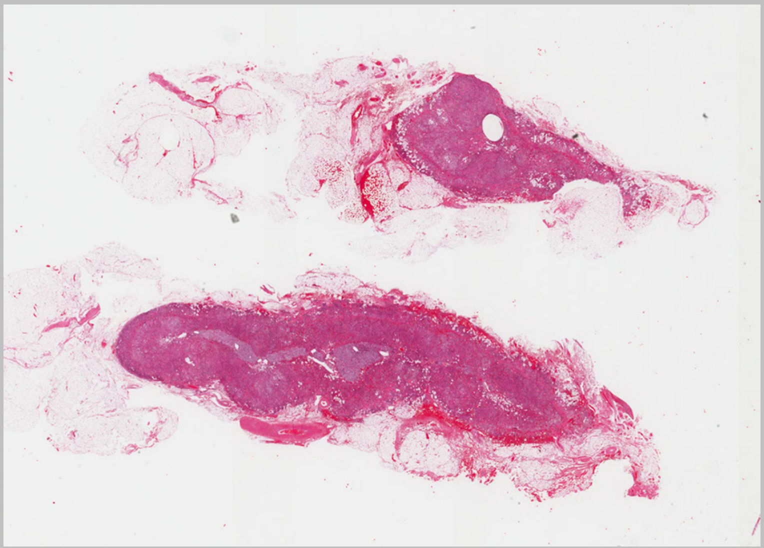

block 1-2, HE, 42714

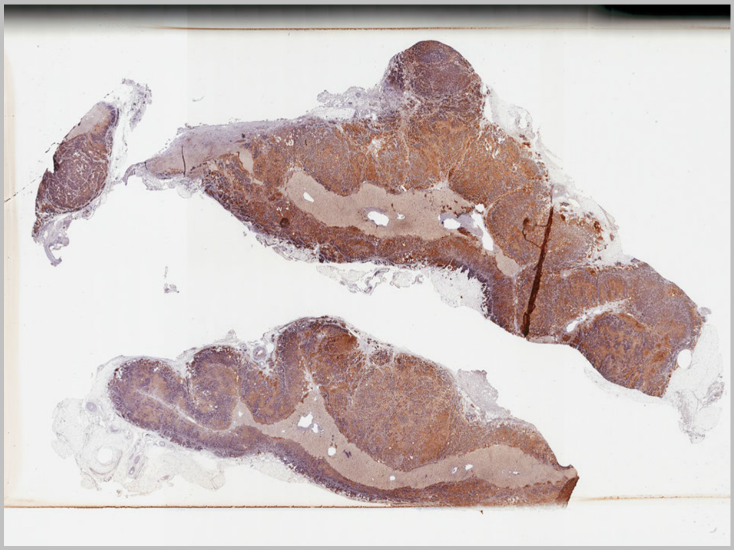

block 3-4, B2B1, 42711

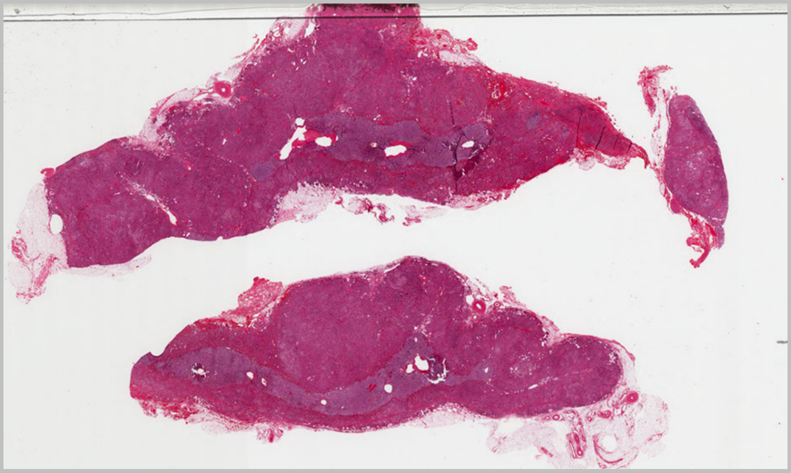

block 3-4, HE, 42712

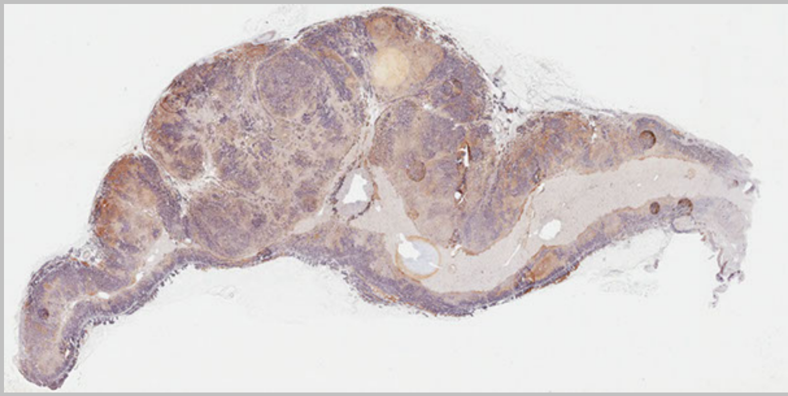

block 5, B2B1, 42795

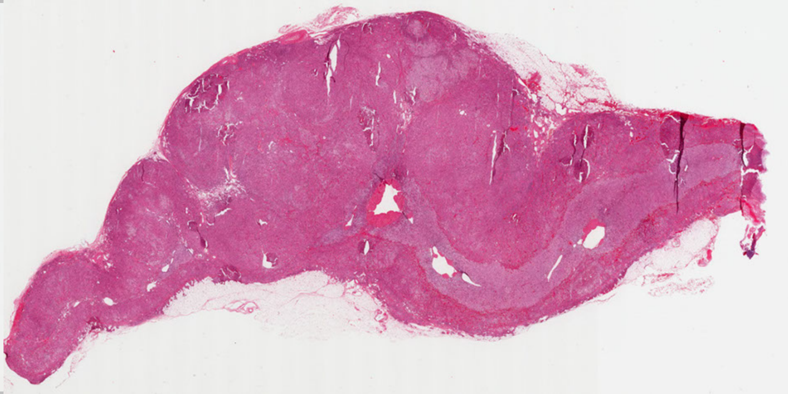

block 5, HE, 42740

Figure S4

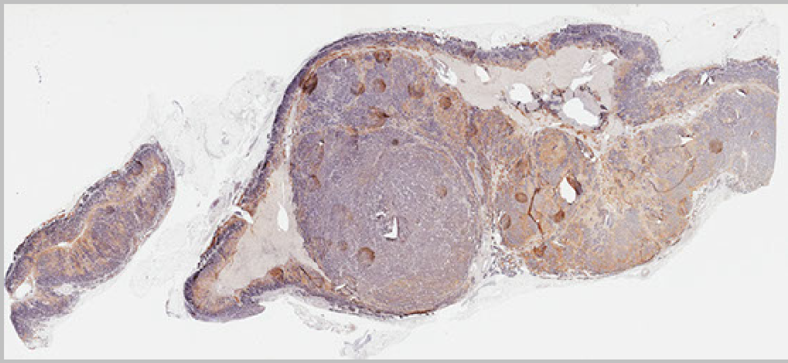

block 6, B2B1, 42737

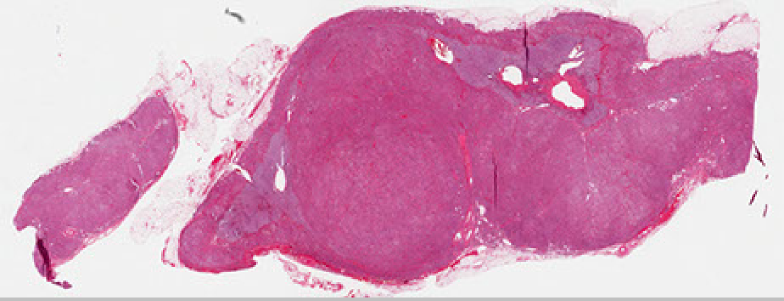

block 6, HE, 42738

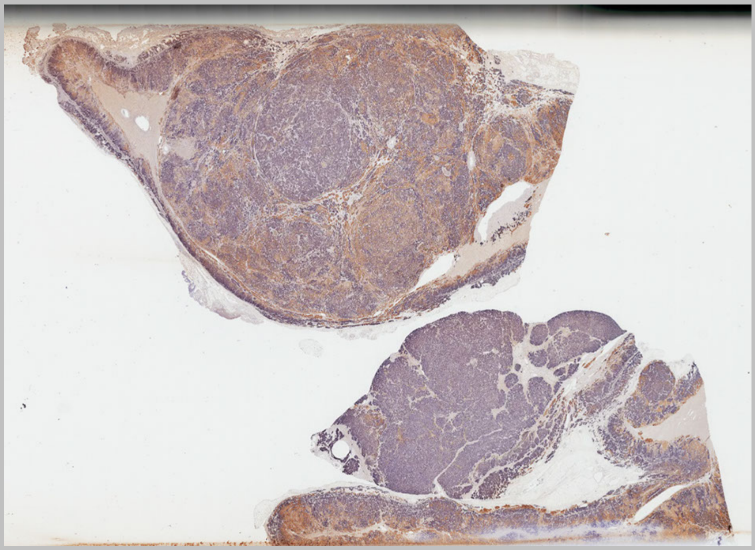

block 7, B2B1, 42735

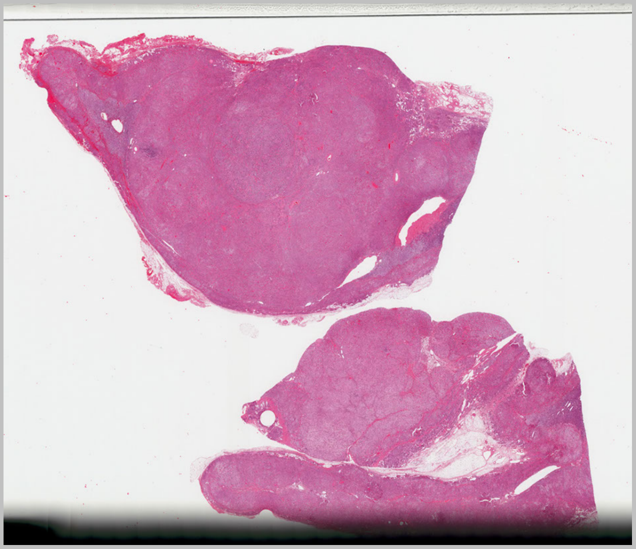

block 7, HE, 42780

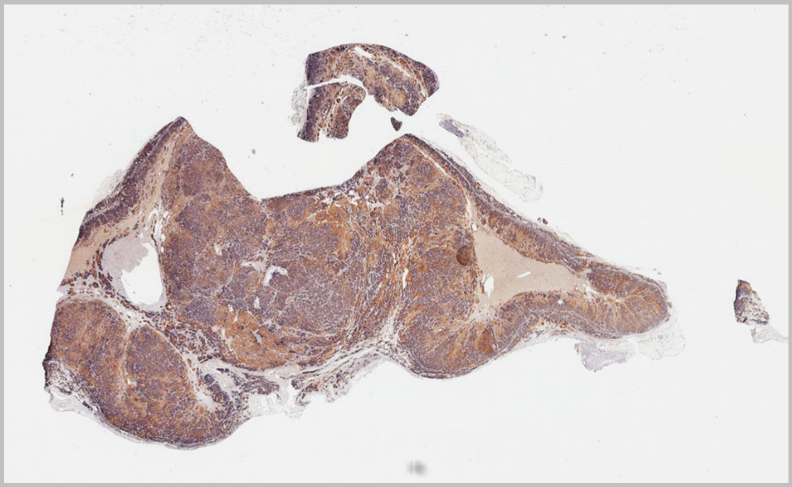

block 8, B2B1, 42778

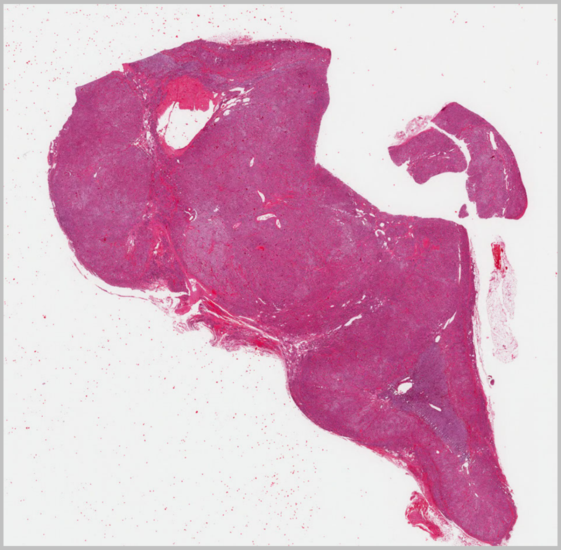

block 8, HE, 42801

Figure S4

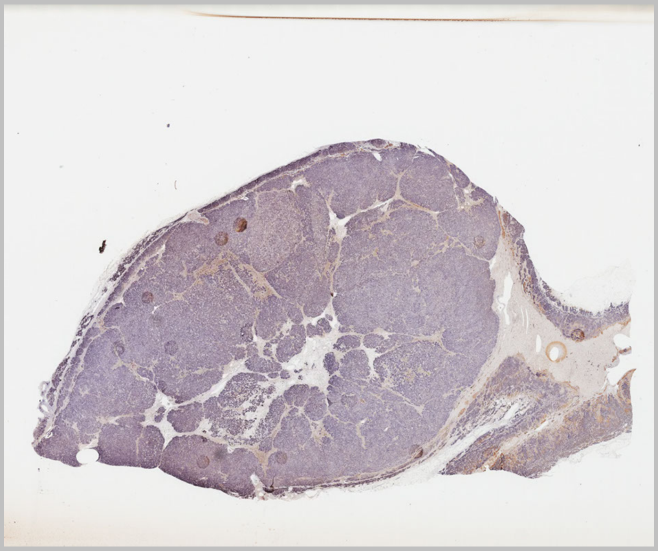

block 9, B2B1, 42731

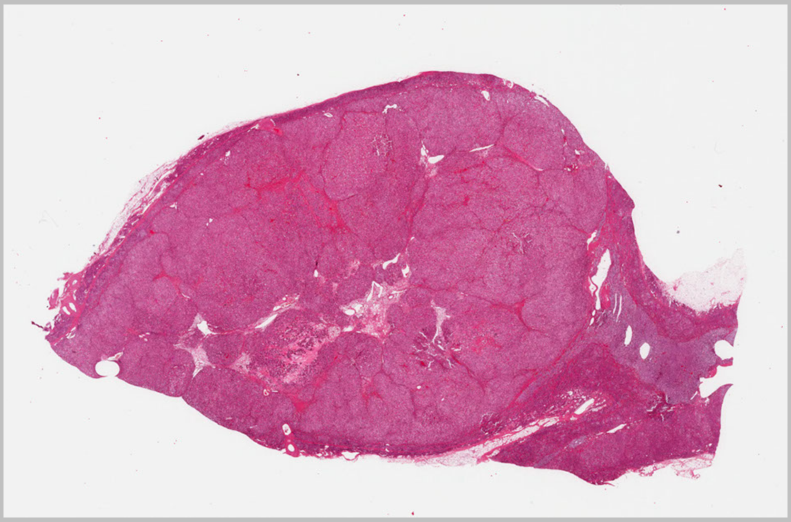

block 9, HE, 42732

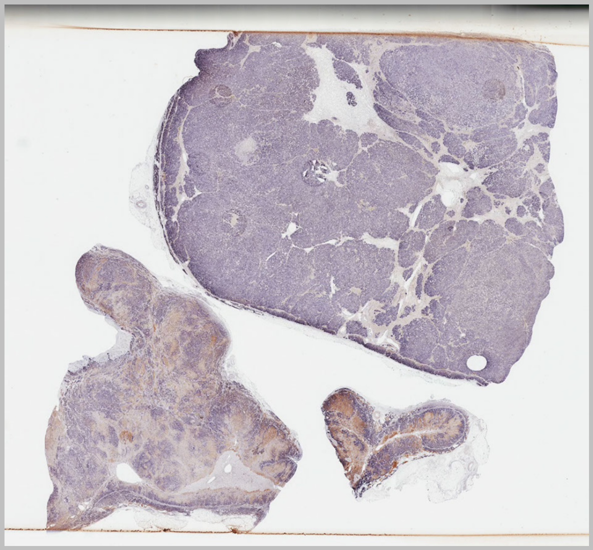

block 10, B2B1, 42729

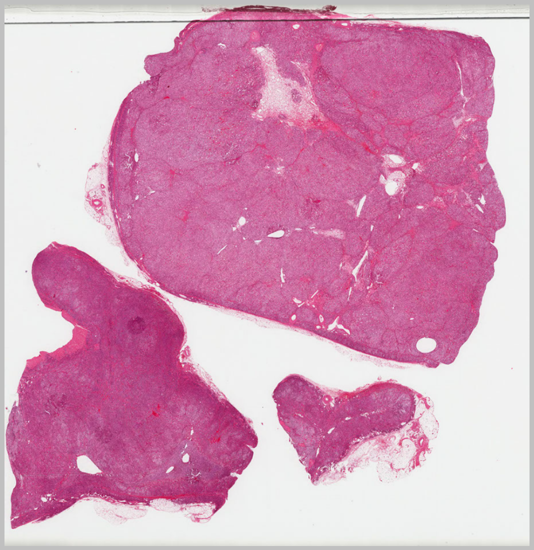

block 10, HE, 42730

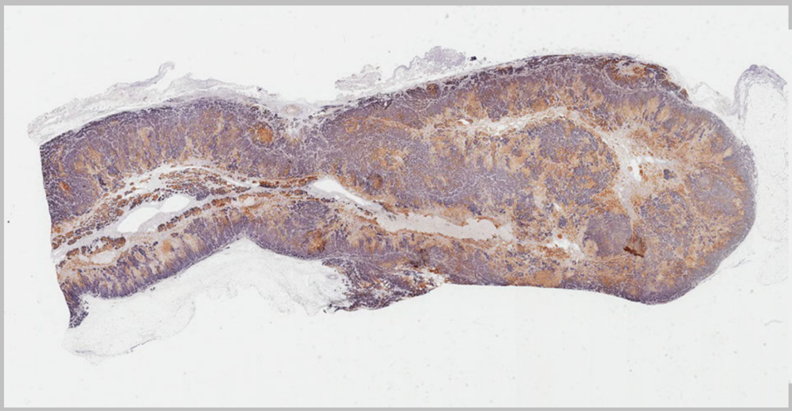

block 11, B2B1, 42727

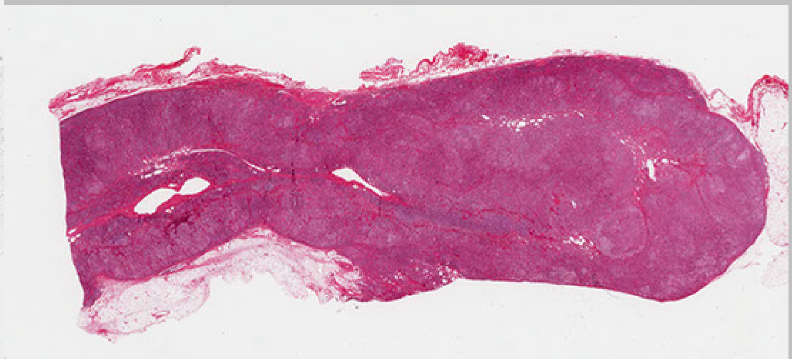

block 11, HE, 42728

Figure S4

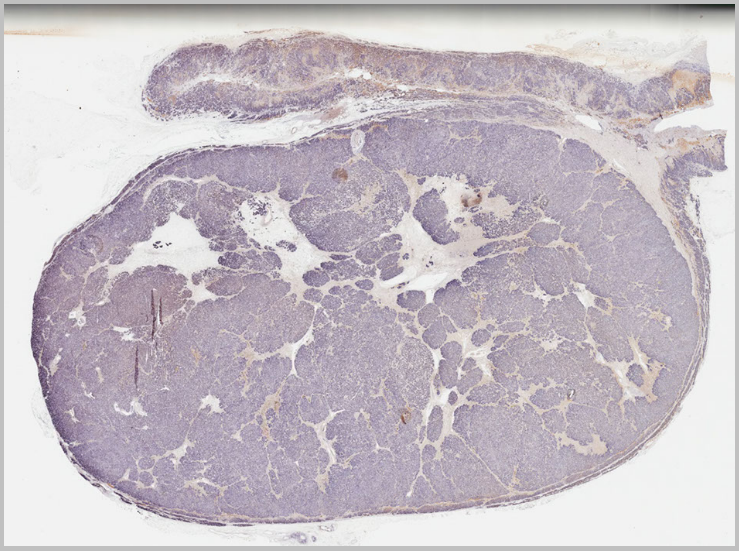

block 12, B2B1, 42755

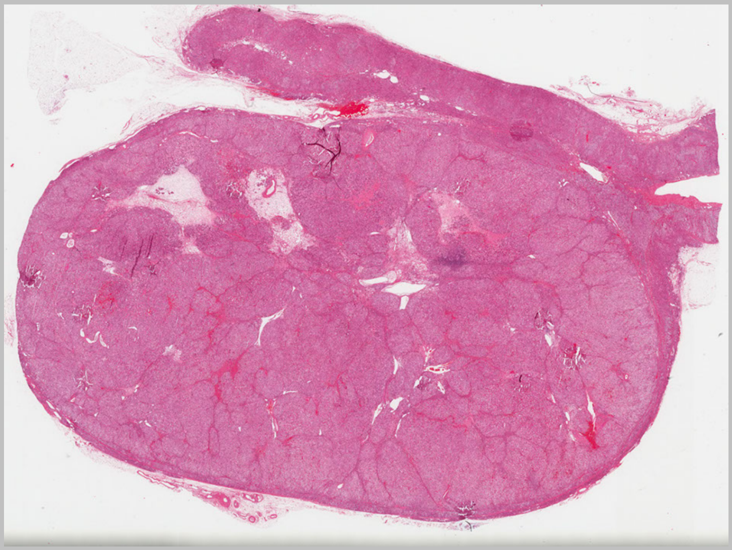

block 12, HE, 42726

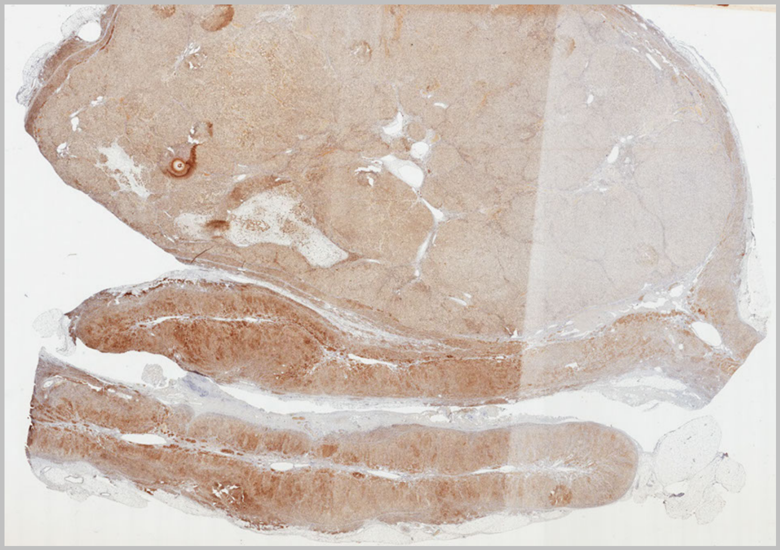

block 13, B1, 42752

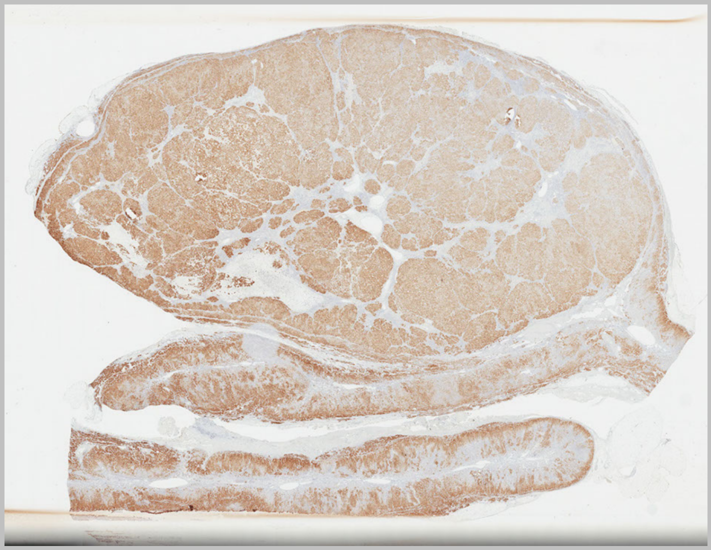

block 13, B2, 42751

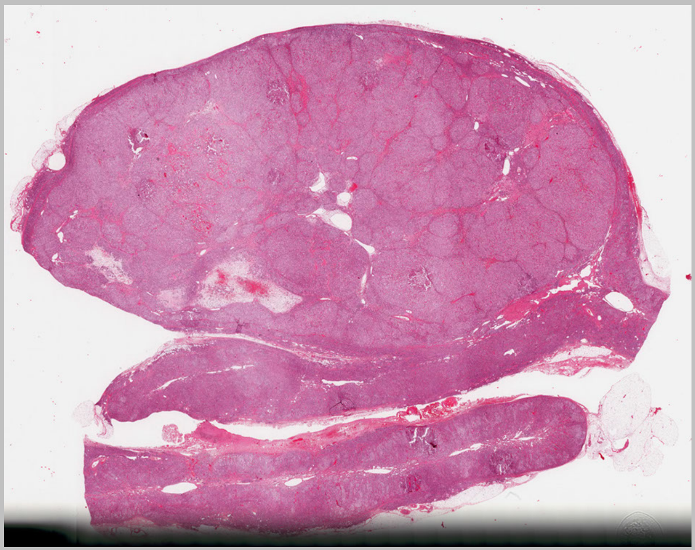

block 13, HE, 42754

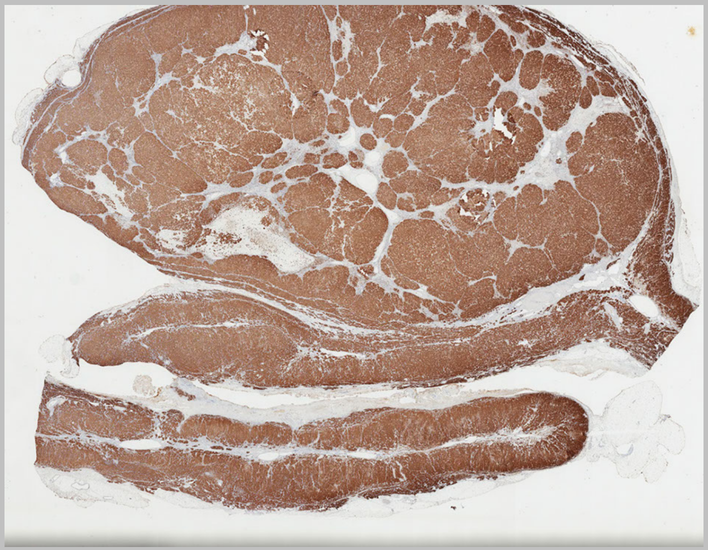

block 13, HSD3B2, 42753

Figure S4

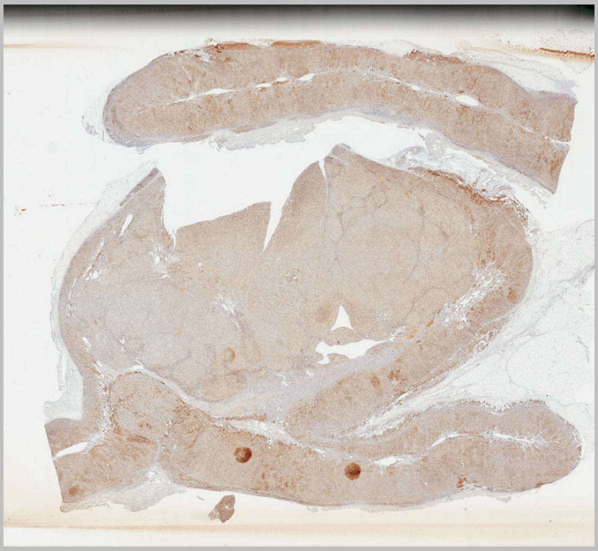

block 14, B1, 42747

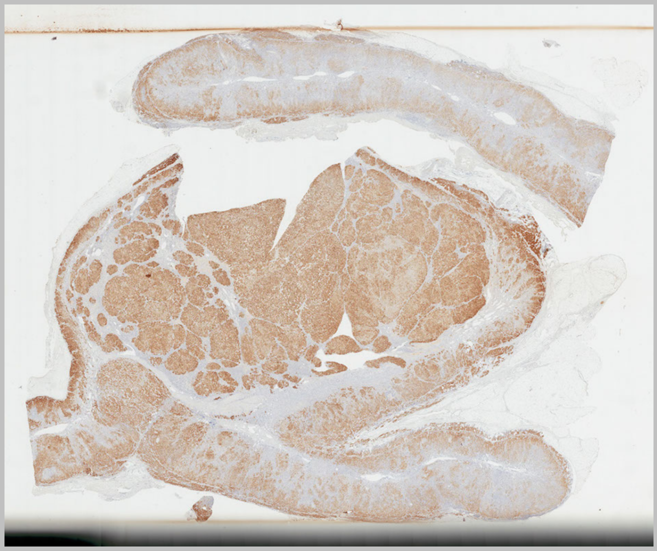

block 14, B2, 42749

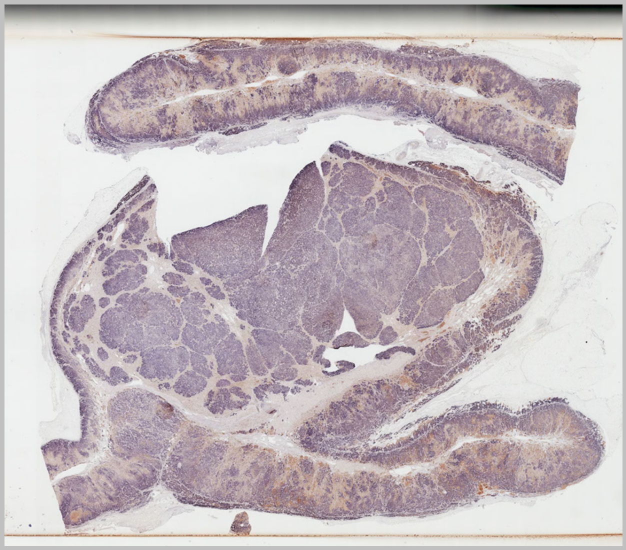

block 14, B2B1, 42746

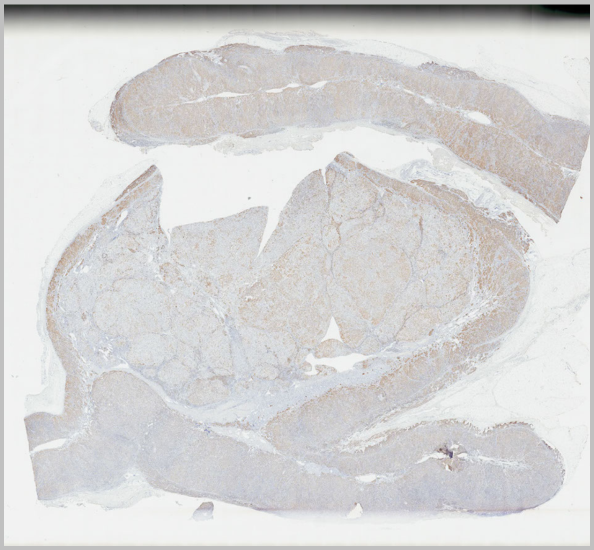

block 14, CYP17A, 42774

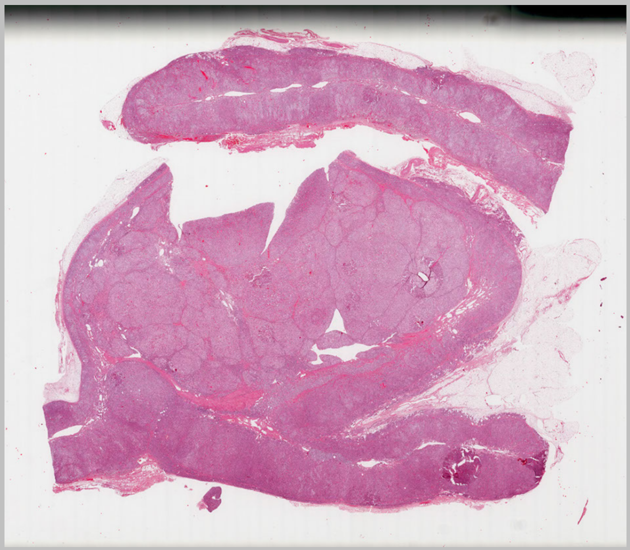

block 14, HE, 42775

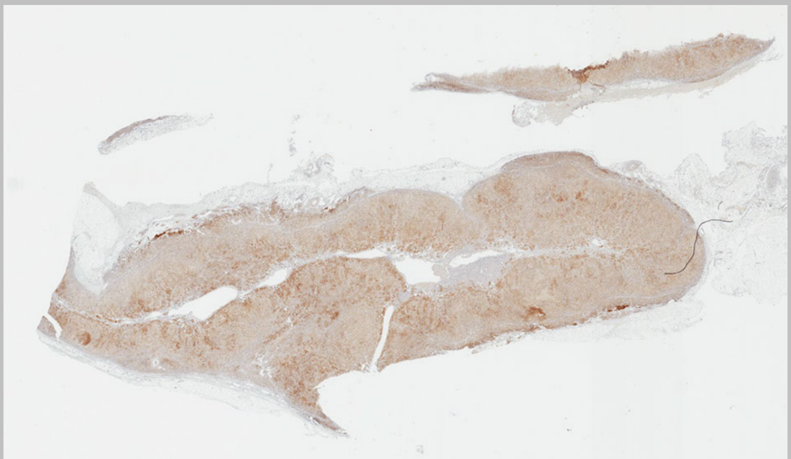

block 15, B1, 42743

Figure S4

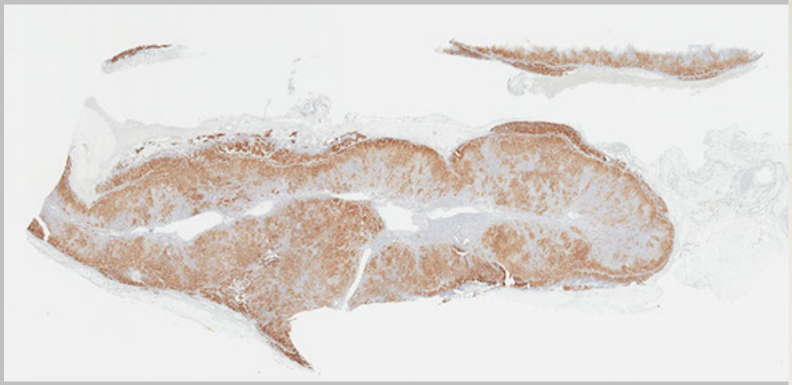

block 15, B2, 42742

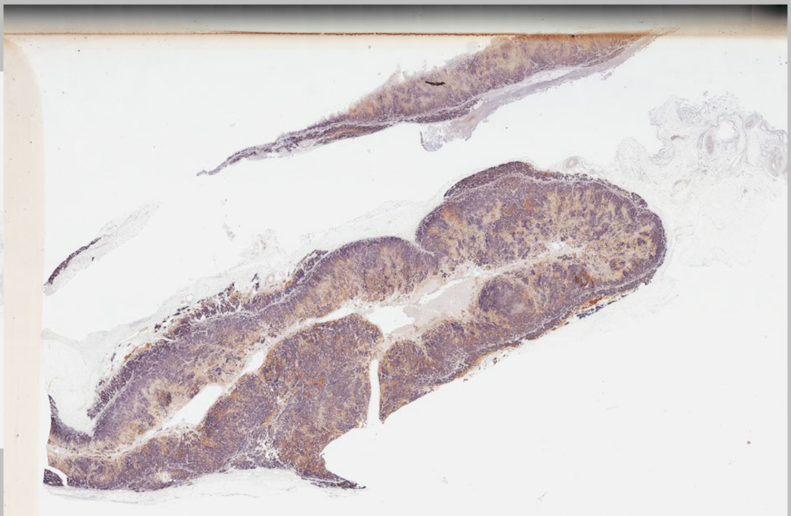

block 15, B2B1, 42744

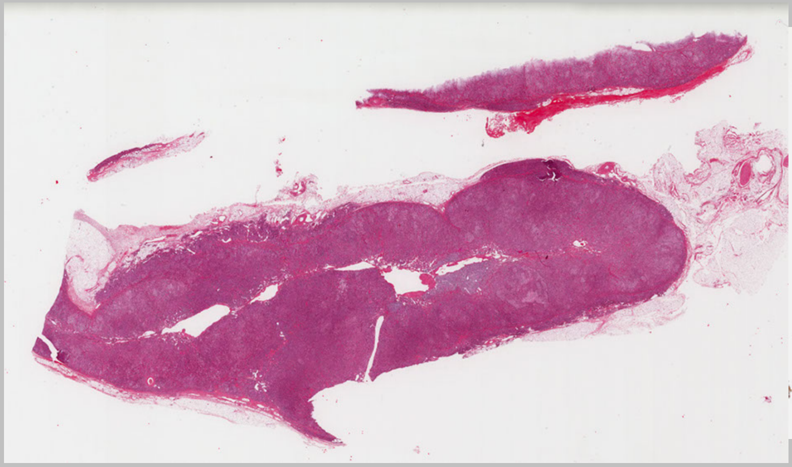

block 15, HE, 42745

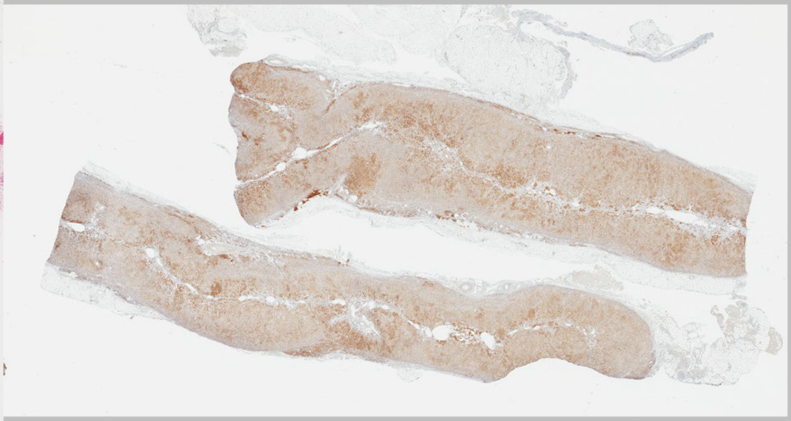

block 16, B1, 42770

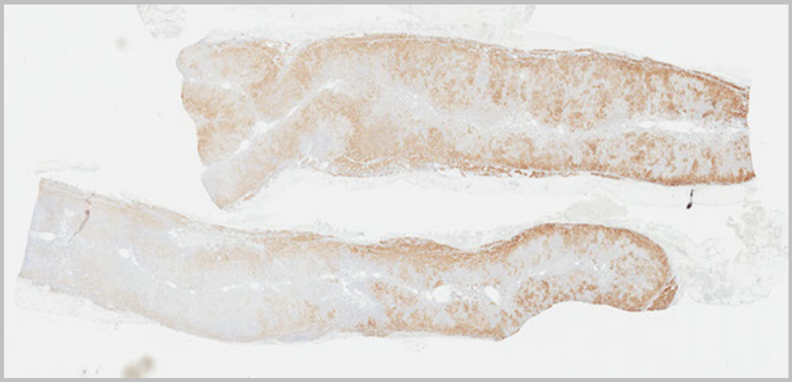

block 16, B2, 42741
