# Supplementary material for: Familial Hyperaldosteronism Type 3 with a Rapidly Growing Adrenal Tumor: An In Situ Aldosterone Imaging Study
Source: Curr Issues Mol Biol. 2021 Dec 28;44(1):128–38. doi: 10.3390/cimb44010010 (PMC8929039; doi:10.3390/cimb44010010)
Supplement: Supplementary file 1 [file cimb-44-00010-s001.zip › Takizawa_et_al._supplemntal_Figure_legends_20211229.pdf]

Supplementary Figure Legends

Figure S1. Formalin-fixed tissues.

Formalin-fixed paraffin-embedded (FFPE) blocks were generated from tissues. Of note, the tissue numbers in Figure 1D–E and the FFPE block numbers in this figure are different; the blue numbers refer to the tissue numbers of the FFPE blocks, while the green numbers in parentheses correspond to the tissue numbers in Figure 1D–E.

Figure S2. Sanger sequencing of the KCNJ5 gene in the index case and her parents

(a): Pedigree of the index case. Her father, mother, brother, and sister did not suffer from primary aldosteronism. (b–d): Sanger sequencing results for KCNJ5 in the adenoma (T1 in Figure 1E), normal adrenal (N1), and peripheral blood from the index case. (e–f): Sanger sequencing results for KCNJ5 in peripheral blood from the father and mother of the index case. Pink frames indicate the mutated portion (p. G151R) of the index case.

Figure S3. Histology of frozen sections

Each image is labeled with the sample name (FB5, FB10, FB15-1, or FB15-2; labeled in Figure 1E), staining method (either immunohistochemistry for CYP11B1 [B1] and CYP11B2 [B2], or hematoxylin-and-eosin staining [HE]), and sample number.

Figure S4. Histology of FFPE sections

Each image is labeled with the sample name (1–2, 3–4, 5, 6, 7, 8, 9, 10, 11, 12, 13, 14, 15, and 16; the block numbers are indicated as blue numbers in Figure S1), staining method (either double immunohistochemistry for CYP11B2 in blue and CYP11B1 in brown [B2B1], single immunohistochemistry in brown for CYP11B2 [B2], CYP11B1 [B1], CYP17A, and HSD3B2, or hematoxylin-and-eosin staining [HE]), and sample number.

Figure S5. Histology of the adrenal cortices of the previously removed left adrenal gland

The adrenal cortices was hyperplastic and had CYP11B2–positive cells with irregular arrangement.

Figure S6. Comparison of *CYP11B2* mRNA levels between this case and the archived cases of sporadic APA (n = 16)

CYP11B2 expression levels were similar in non-tumor (816,286.5 ± 428,233.7-fold) and tumor portions (539,737.3 ± 336,381.8-fold) of the case (p=0.393, Unpaired Student's t-test ΔΔCt values). *CYP11B2* expression levels in the case (T1–T3 and N1–N3, 582,237 [interquartile range: 448,734–977,189] fold) and APA (574,401 [328,933–817,145] fold)

were significantly higher than that of the paired adjacent normal adrenals (966 [62–11,986] fold), and those in the case and APA were similar. *$p < 0.05$

Figure S7. Immunohistochemistry for KCNJ5

Immunostaining of KCNJ5 in a normal adrenal gland from a renal cell carcinoma patient (SIMC–Uro#10635, left) and a frozen section from FB10 (right). Three portions in red frames in the subcapsular area of the normal adrenal, non-tumor portion of FB10, and tumor portion of FB10 are enlarged in panels B, C, and M, respectively.

Figure S8. Immunohistochemistry for CTNNB1

Immunostaining of CTNNB1 from frozen sections of FB10 (right). The two portions indicated by yellow arrowheads are enlarged in panels B and C. Blue and green arrowheads indicate positive and negative nuclear staining for CTNNB1, respectively.
