# Supplementary figures and images for: Familial Hyperaldosteronism Type 3 with a Rapidly Growing Adrenal Tumor: An In Situ Aldosterone Imaging Study
Source: Curr Issues Mol Biol. 2021 Dec 28;44(1):128–38. doi: 10.3390/cimb44010010 (PMC8929039; doi:10.3390/cimb44010010)

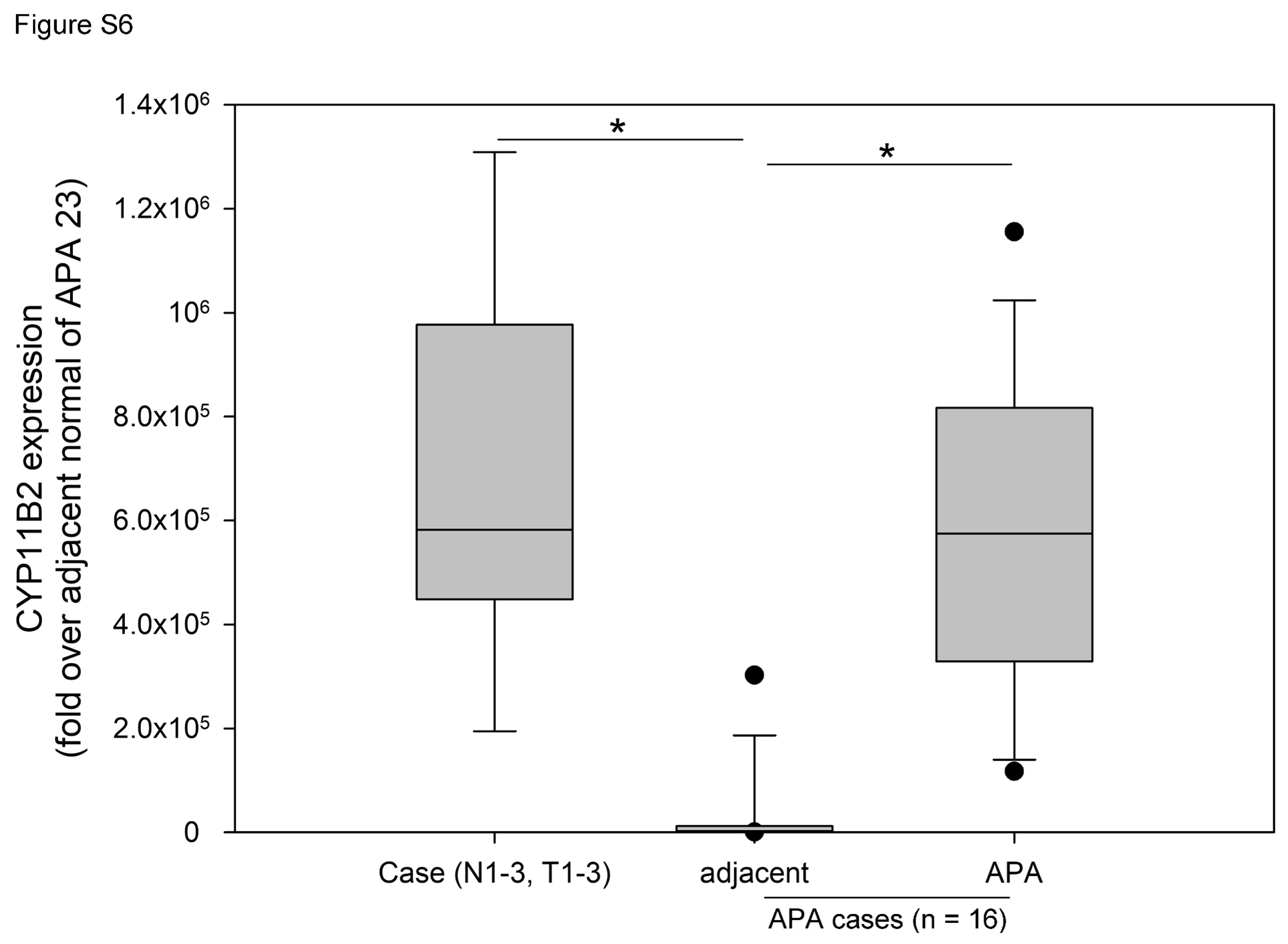

Supplement: Supplementary file 1 [file cimb-44-00010-s001.zip › Figure S6.tif]

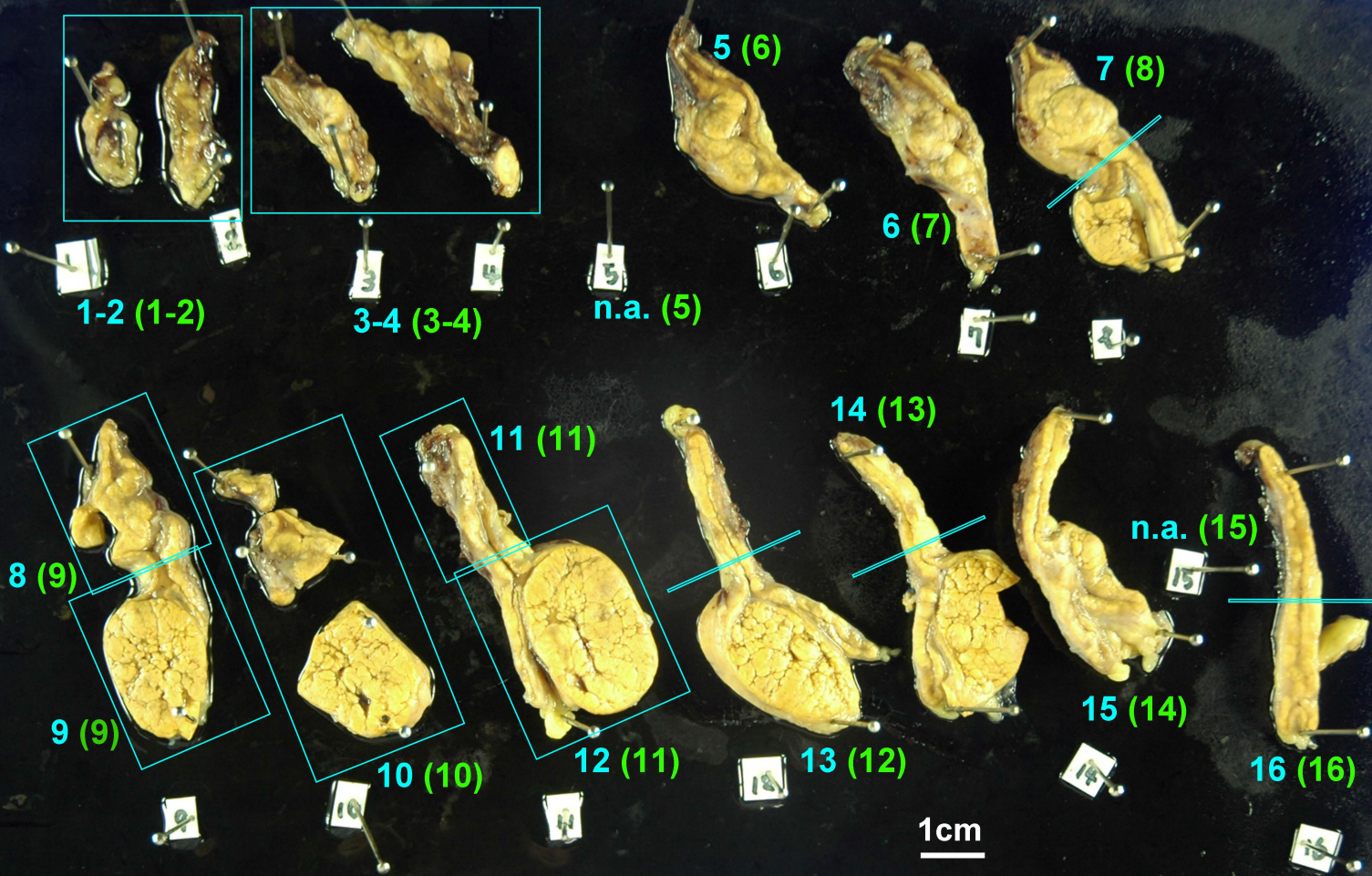

Supplement: Supplementary file 1 [file cimb-44-00010-s001.zip › Figure_S1_FFPE blocks.pdf]

Figure S3

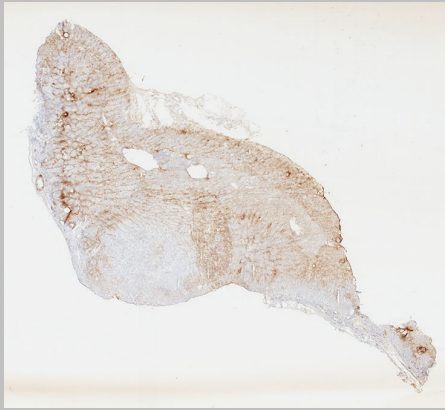

FB5, B1, 42764

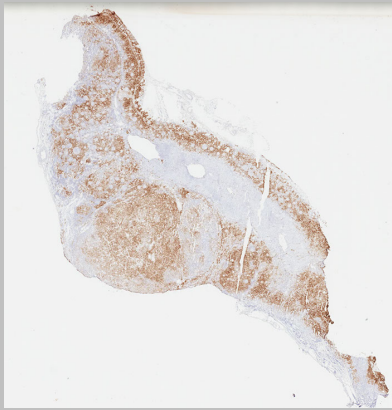

FB5, B2, 42763

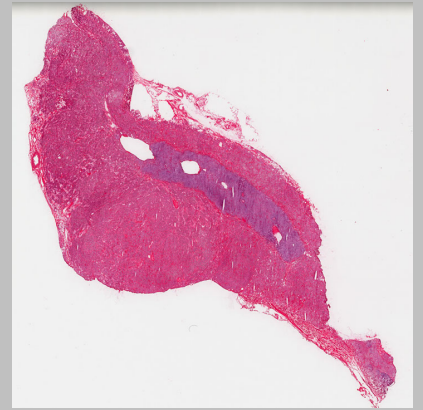

FB5, HE, 42765

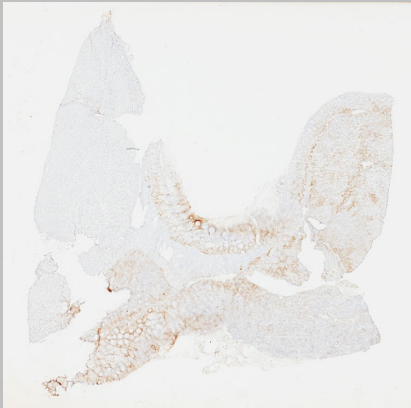

FB10, B1, 42721

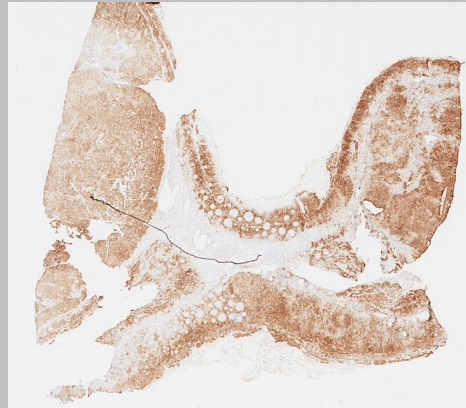

FB10, B2, 42722

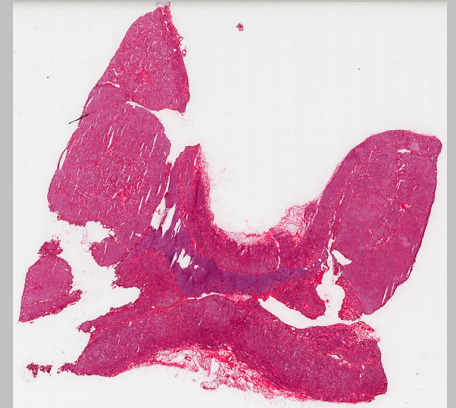

FB10, HE, 42723

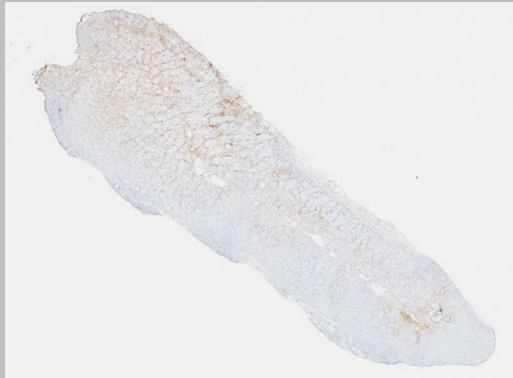

FB15-1, B1, 42784

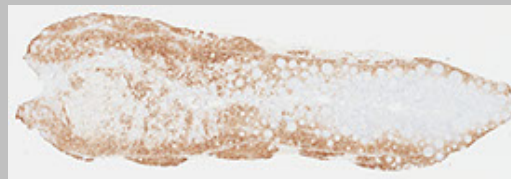

FB15-1, B2, 42719

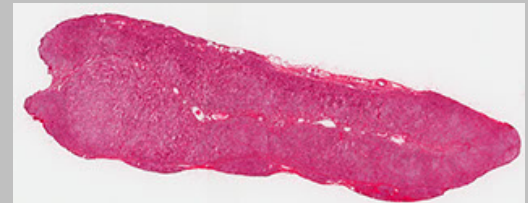

FB15-1, HE, 43221

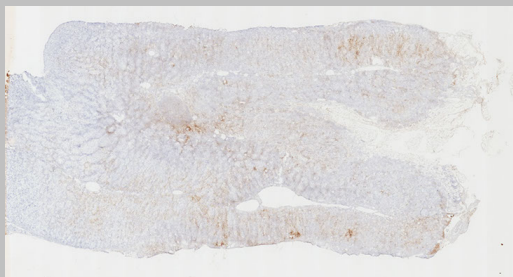

FB15-2, B1, 42715

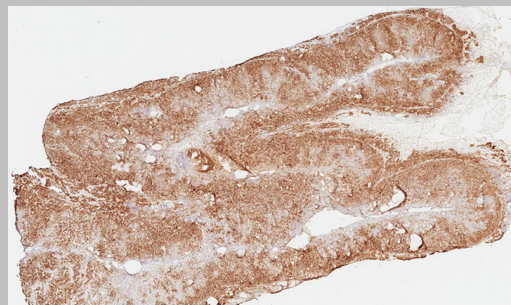

FB15-2, B2, 42716

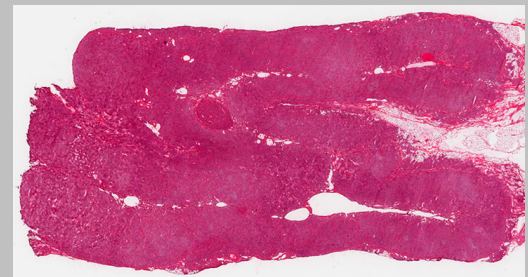

FB15-2, HE, 42717

Supplement: Supplementary file 1 [file cimb-44-00010-s001.zip › Figure_S3_Data 1_frozen.pdf]

**Figure S5**

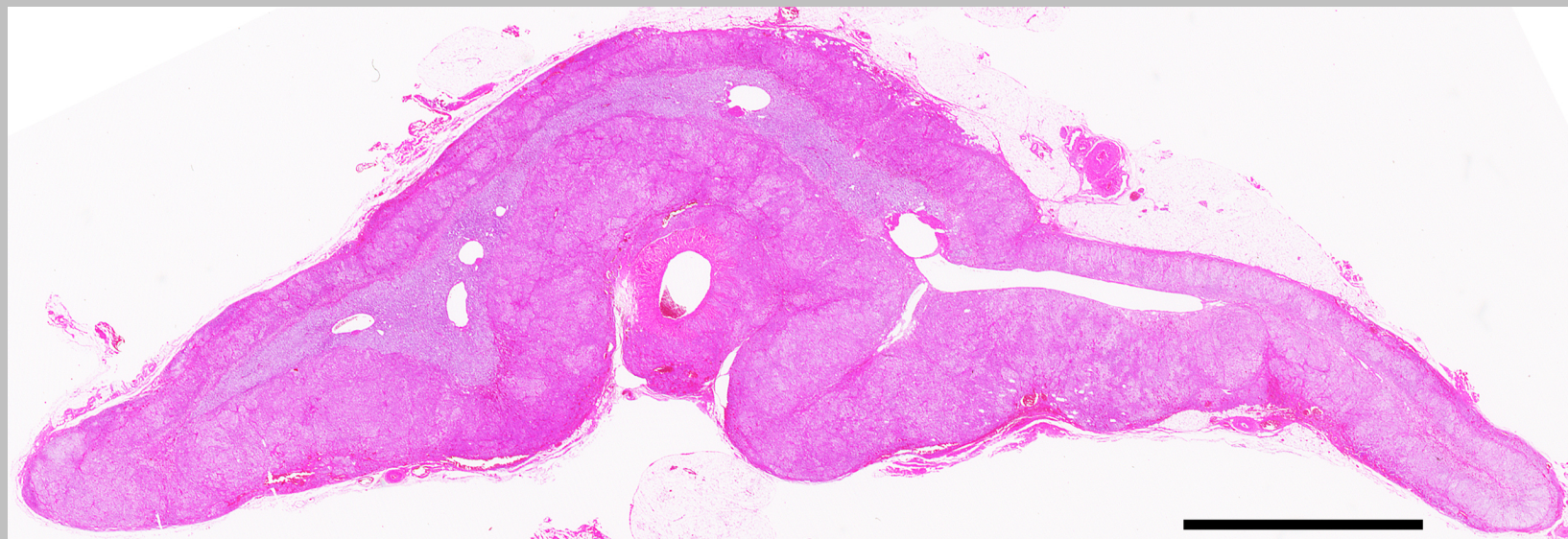

Left adrenal, H&E

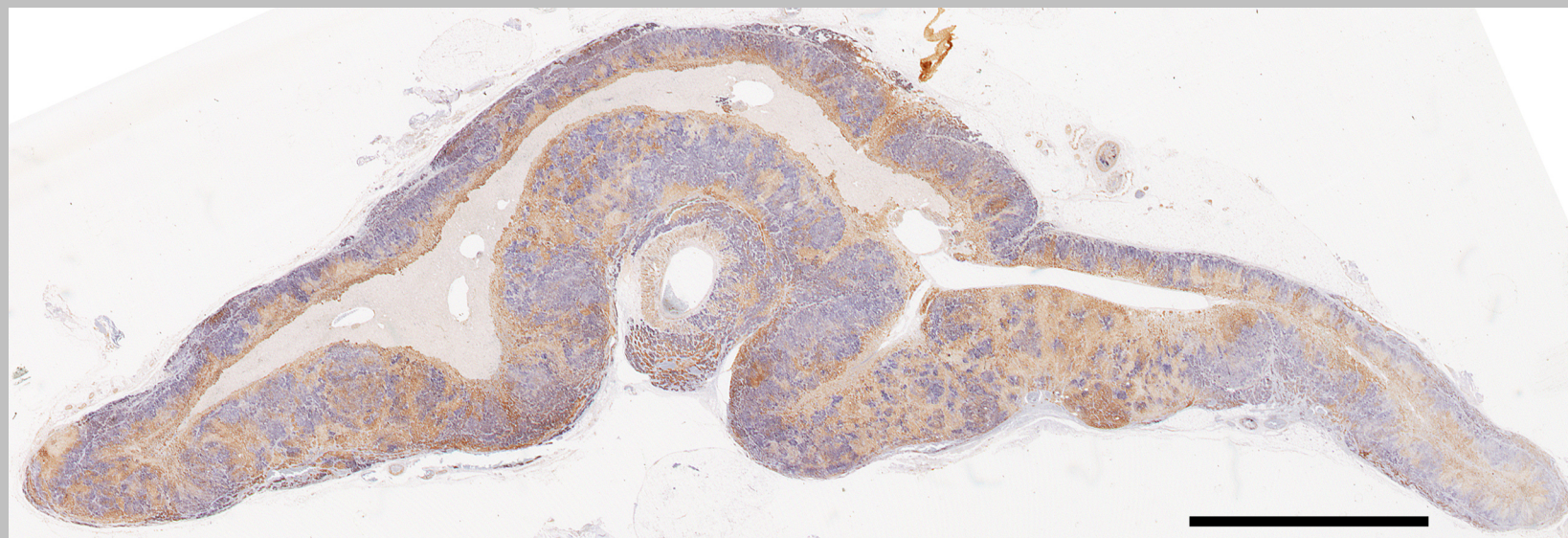

Left adrenal, CYP11B2 & CYP11B1

bars: 5 mm

Supplement: Supplementary file 1 [file cimb-44-00010-s001.zip › Figure_S5_previous adrenal.pdf]

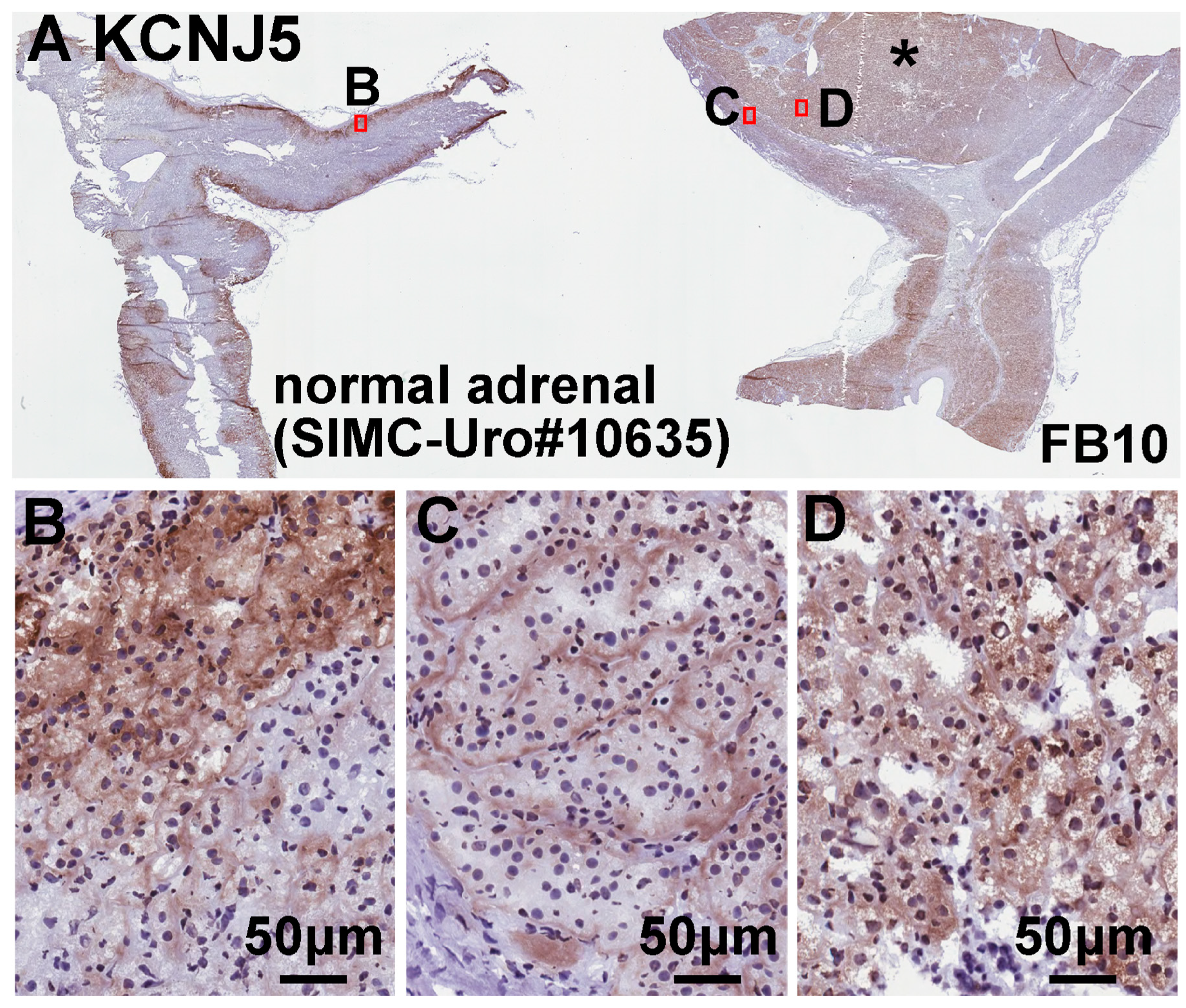

Supplement: Supplementary file 1 [file cimb-44-00010-s001.zip › Figure_S7, KCNJ5.tif]

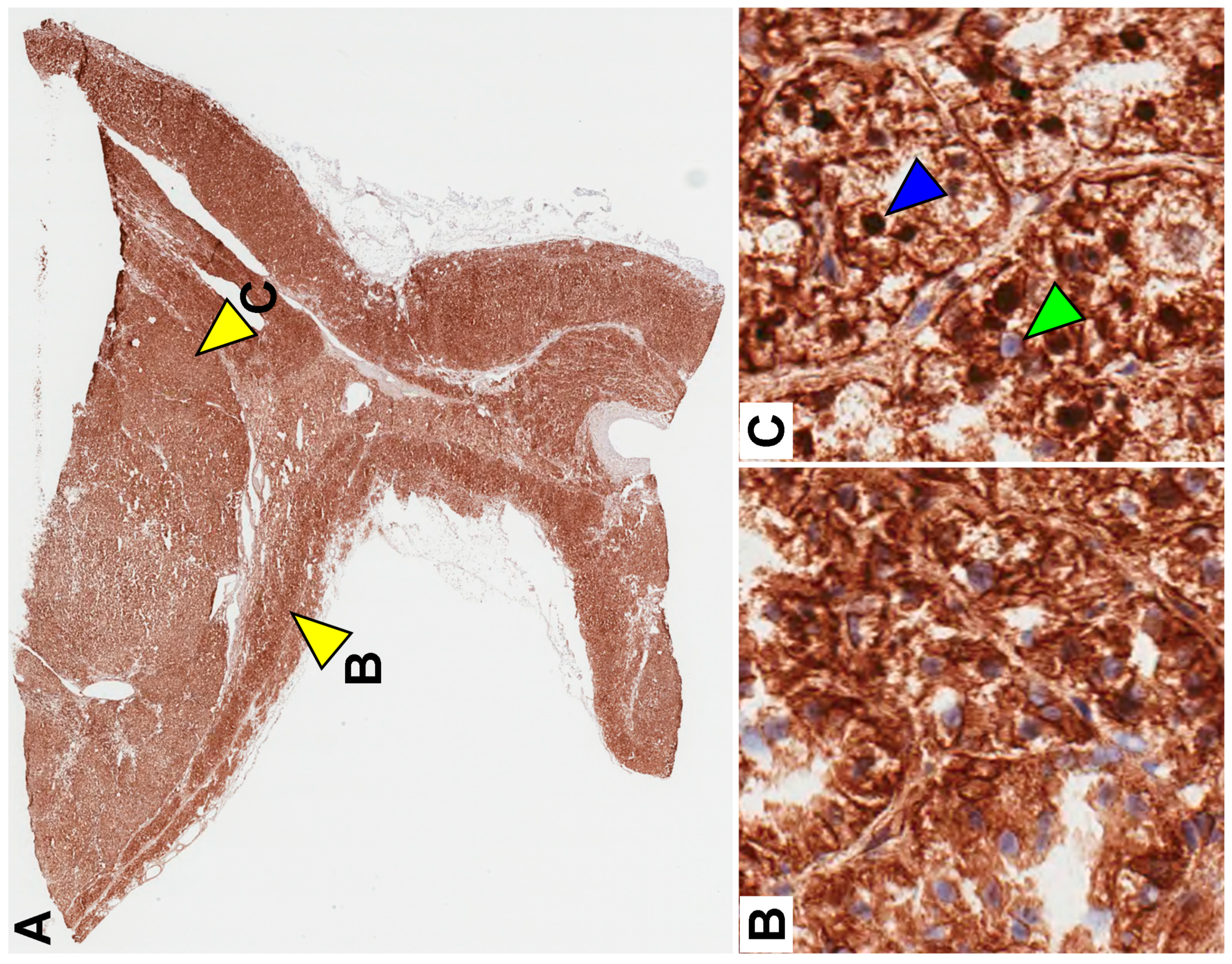

Supplement: Supplementary file 1 [file cimb-44-00010-s001.zip › Figure_S8.tif]
